# Effect of resistance exercise on physical fitness, quality of life, and fatigue in patients with cancer: a systematic review

**DOI:** 10.3389/fonc.2024.1393902

**Published:** 2024-07-19

**Authors:** Qiuhua Zhang, Yanan Gao, Wenjun Wang, Xiaoguang Zhao, Jiabin Yu, Huiming Huang

**Affiliations:** ^1^ Faculty of Sports Science, Research Academy of Grand Health, Ningbo University, Ningbo, Zhejiang, China; ^2^ School of Physical Education and Sport Science, Fujian Normal University, Fuzhou, Fujian, China; ^3^ Ningbo New Fitness Health Technology Co., Ltd, Ningbo, Zhejiang, China

**Keywords:** resistance exercise, cancer, physical fitness, QOL, fatigue, systematic review

## Abstract

**Objective:**

The purpose of this study is to conduct a systematic review to assess the effects of different forms of resistance exercises (resistance exercise, resistance exercise combined with aerobic exercise, and resistance exercise combined with other exercises) on physical fitness, quality of life (QOL), and fatigue of patients with cancer.

**Methods:**

We conducted a systematic review using the Cochrane Handbook for Systematic Reviews of Interventions guidelines. We searched PubMed, Web of Science, and Scopus databases for the studies from the establishment of the database to September 2023, including randomized controlled trials and clinical trials that evaluated the effects of different resistance exercise on physical fitness, QOL, and fatigue in all patients with cancer. Two reviewers independently assessed the quality of all the included studies using the Cochrane Handbook for Systematic Reviews of Interventions and MINORS scale. We divided the intervention into three types: resistance exercise, resistance exercise combined with aerobic exercise, and resistance exercise combined with other exercises.

**Results:**

In total, 48 studies (3,843 participants) met the inclusion criteria. The three exercise intervention forms have significant effects on physical fitness and QOL, but the improvement effect on fatigue is not clear. A total of 34 studies reported significant and beneficial effects of resistance exercise on physical fitness across all types of cancer. There were 28 studies that reported significant or borderline improvement effects of resistance on QOL, and only 10 studies reported significant effects of resistance exercise interventions on fatigue improvement in patients with cancer.

**Conclusions:**

Resistance exercise, resistance exercise combined with aerobic exercise, and resistance exercise combined with other exercises all have a positive effect on improving fitness and QOL in patients with cancer. Resistance exercise has an advantage in improving muscle strength, while combined resistance exercise has an advantage in improving QOL; however, there are no consistent findings in improving fatigue, although low-intensity resistance exercise is effective.

**Systematic review registration:**

www.inplasy.com, identifier INPLASY2023110034.

## 1 Introduction

Cancer is the second leading cause of death worldwide (1). As the number of cancer survivors and their longevity increase, long-term health issues related to cancer and its treatment are becoming more critical (2). However, patients with cancer receiving treatment often suffer from nausea, insomnia, diarrhea, and other treatment-related symptoms and side effects (3). Regardless of their cancer type, patients report decreased physical fitness (4, 5), fatigue, and reduced quality of life (QOL) (6). Consequently, there is growing interest in the impact of exercise interventions on patients with cancer. Increasing evidence shows that exercise interventions can significantly improve the physical and psychological functioning of patients with cancer, including their QOL (7, 8). Although the American College of Sports Medicine (ACSM) recommends exercise during cancer treatment, it does not provide specific guidelines on which type and intensity of physical exercise are most effective during chemotherapy or treatment (9).

Evidence suggests that moderate weekly physical activity improves cancer survivors’ QOL, physical activity levels, physical fitness, body mass index (BMI), and hormone levels (10–12), However, research on the effects of resistance exercise on cancer is still limited. Most systematic reviews to date have focused on breast cancer research. One study showed that guideline-compliant aerobic exercise can reduce breast cancer mortality and all-cause mortality compared with patients who do not meet physical activity guidelines (13). A 2013 meta-analysis assessed the effect of exercise training on the QOL of breast cancer survivors (14), but this analysis only included randomized controlled trials (RCTs) up to 2013. Zhang et al. conducted a comprehensive review of the impact of exercise on the QOL of patients with breast cancer, including various exercise interventions, but limited to breast cancer and a single QOL indicator (15). Although there was a study on the effect of exercise intervention on all types of cancer, it did not specify the effects on specific cancers and included fewer indicators in the results (16). Neo- and adjuvant therapies have increased the survival rate among patients with cancer (17, 18). However, as life expectancy increases, the side effects of long-term treatment persist and significantly impact the health and QOL of patients with cancer. Consequently, adverse effects on physical health indicators, including cardiorespiratory fitness and strength, are often observed clinically (19). Chemotherapy or radiation therapy often leads to dysfunction and reduced QOL in patients with cancer. Exercise has been shown to improve factors such as functional capacity and QOL in patients undergoing chemotherapy, therapy, or combination therapy (20). Increasing evidence suggests that regular physical activity, such as 3–5 h of moderate-intensity walking per week, reduces the risk of cancer-specific and all-cause mortality by 30%–50% compared with inactive patients with cancer (21). However, many studies on the effects of exercise programs on patients with cancer during radiation therapy have focused primarily on breast cancer cases (22). Fewer studies have investigated structured exercise-based rehabilitation programs to improve fitness and QOL in patients with various types of cancer, as well as programs combining resistance training with aerobic and other exercises. Recently, there has been a gradual increase in experimental studies of exercise interventions for patients with cancer. The effects of aerobic exercise on cancer are well established, and resistance training is the only known non-drug intervention that can improve skeletal muscle quality, strength, and prevent muscle loss (23). Therefore, combining resistance exercise with aerobic exercise has been proposed as a multidimensional intervention for patients with cancer to explore new methods of exercise-based therapy (24). However, evidence from experimental studies on resistance exercise interventions in patients with cancer is still limited, and some results are controversial. For example, some studies have found significant improvements in the QOL of patients with cancer after resistance exercise interventions (25–28), while others have reached different or even opposite conclusions (29–31). Although exercise has been proven to be effective in the treatment and rehabilitation of patients with cancer, comparison between different types of exercise and cancer is still relatively lacking.

Therefore, we conducted a systematic review of all experimental studies to date with the aim of exploring the effects of resistance exercise, resistance exercise combined with aerobic exercise, and resistance exercise combined with other exercises on physical fitness, QOL, and fatigue in patients with cancer. Our goal is to provide a new reference value for non-drug intervention in the treatment of clinical patients with cancer.

## 2 Methods

### 2.1 Registration number

We registered the review protocol in INPLASY (registration number INPLASY2023110034) and adhered to the Preferred Reporting Items for Systematic Reviews and Meta-Analyses (PRISMA) 2020 guidelines and synthesis without meta-analysis guidelines.

### 2.2 Search strategy

We conducted comprehensive searches in PubMed, Web of Science, Scopus, and other databases comprehensively, and retrieved other potentially relevant studies from the included references. We included all published English literature from the inception of each database to 1 September 2023.

Our search strategy included Medical Subject Headings (Mesh) related to cancer and resistance training, along with free text terms (**Supplementary Table 1**).

Two authors independently conducted the retrieval. All potentially relevant studies meeting the predetermined inclusion criteria were included in the review. The third author resolved any disagreement arising in the retrieval process.

### 2.3 Eligibility and excluded criteria

#### 2.3.1 Types of studies

The included studies comprised RCTs and clinical intervention trials (non-RCT), including self-control and other quasi-experimental studies, with the language limited to English. Animal experiments and observational studies were excluded.

#### 2.3.2 Participants

Participants were adults diagnosed with cancer, either undergoing treatment or having completed treatment, and older than 18 years old. Patients with severe cardiovascular disease, sports system disease, or weakness that prevented participation in the exercise program were excluded.

#### 2.3.3 Interventions and comparator

The inclusion criteria were as follows: (1) the intervention group received resistance exercise intervention, resistance exercise combined with aerobic exercise, or resistance exercise combined with other forms of exercise. (2) The control group did not receive any form of exercise intervention, including blank control and diet control. (3) Studies with a single-group pre- and post-control design, where exercise training is part of multiple interventions (e.g., combined with protein supplementation), were not excluded. Studies in which exercise was part of the control group were excluded.

#### 2.3.4 Outcomes

The results of these included studies require reporting physical fitness, fatigue, QOL, or one of the outcome indicators. Studies that did not report any of these outcomes were excluded.

### 2.4 Data extraction

Two authors searched the aforementioned electronic database using the developed retrieval strategy. The titles and abstracts of all retrieved articles were independently screened by the two authors, and duplicates were removed. Then, according to the inclusion criteria, two authors independently reviewed the full text of articles that might be included in the study. All disputes arising in this process were settled by the third author through consultation. Finally, we extracted data from the selected studies using a predefined table. The extracted data mainly included the following: author, year, country, study name, study design, study population, sample size, mean age, proportion of female patients, type of exercise, frequency of exercise, duration of exercise, intensity of exercise, measurement instruments, comparative results, and description of results.

### 2.5 Risk of bias of evidence

Two authors independently completed the quality assessment of the included studies. The quality assessment of RCTs followed the Cochrane Handbook for Systematic Reviews of Interventions (32). The quality evaluation criteria included seven parts: random sequence generation, distribution hiding, blinding of participants, blinding of researchers, blinding of result evaluation, incomplete result data, and selective report of results and other biases. According to the Cochrane Handbook, each evaluation item was classified into three levels: low risk, unclear risk, and high risk. The purpose was to evaluate selection, performance loss, and detection deviation through the possible bias risk of RCT design, trial, and outcome evaluation, and to understand the internal authenticity of the experimental study. The quality of non-RCTs was evaluated according to the MINORS criteria (33), which include 12 evaluation items, each scored from 0 to 2 points. The highest score of the first eight items for the study without the control group was 16 points; The last four items and the first eight items are for the study with the control group, and the maximum score is 24 points in total. 0 indicates no report; 1 point means that the report is reported but the information is not sufficient; 2 points means that the report has been reported and sufficient information has been provided.

### 2.6 Analysis

Because of the heterogeneity of measurement tools in the included studies, the expression of the outcome indicators was inconsistent; thus, a meta-analysis was not performed. Therefore, in accordance with the Cochrane Handbook for Systematic Reviews of Interventions, a systematic evaluation and qualitative analysis were conducted on the included studies.

**Figure 1:**
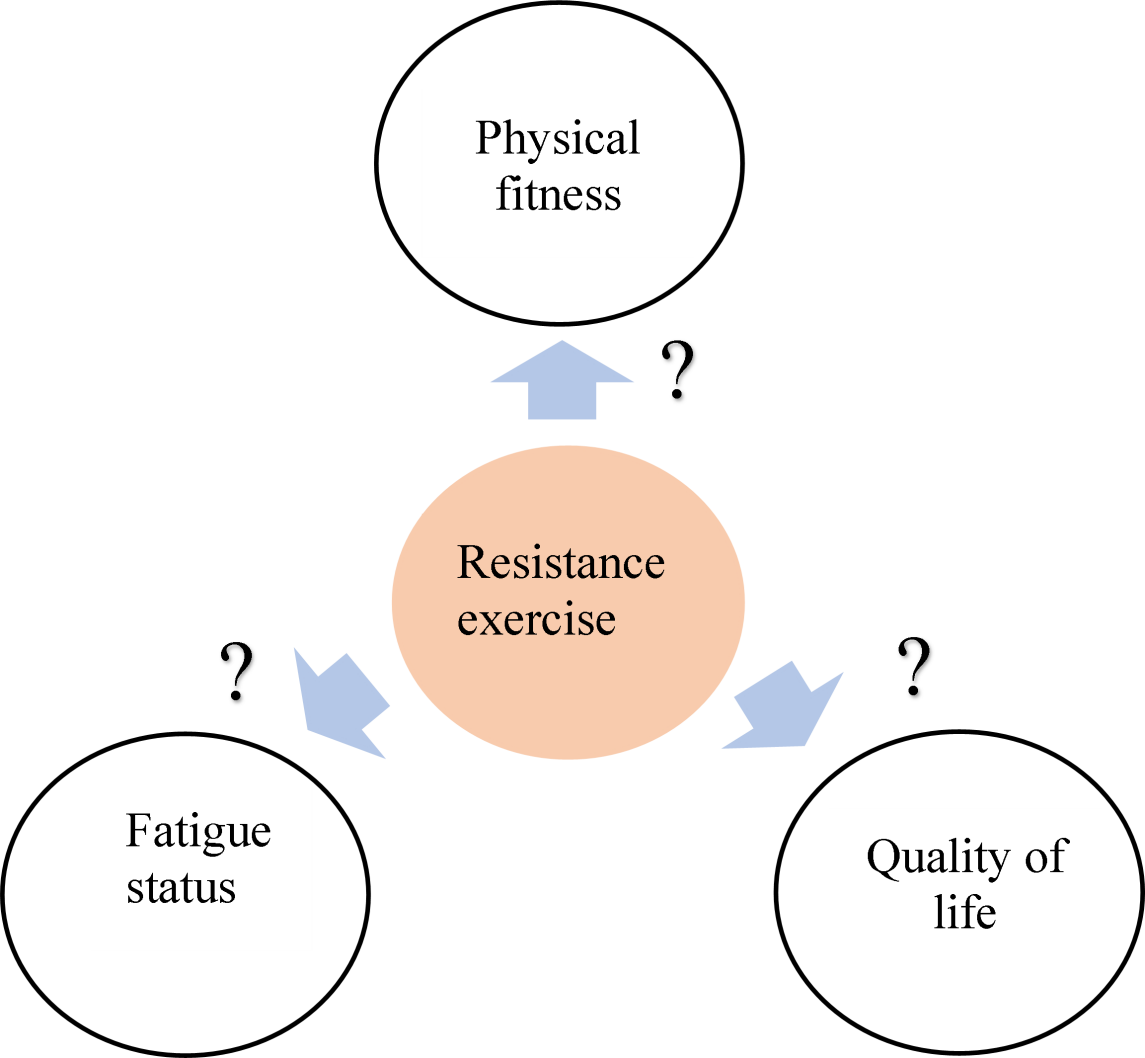
A conceptual framework for the impact of resistance training on patients with cancer.

We categorized studies according to three outcomes—physical fitness, QOL, and fatigue. **Figure 1** described changes in relevant indicators after resistance training intervention. The research under each outcome was categorized into three types of exercise: resistance exercise, resistance exercise combined with aerobic exercise, and resistance exercise combined with other exercises.

## 3 Results

### 3.1 Study search and selection

**Figure 2:**
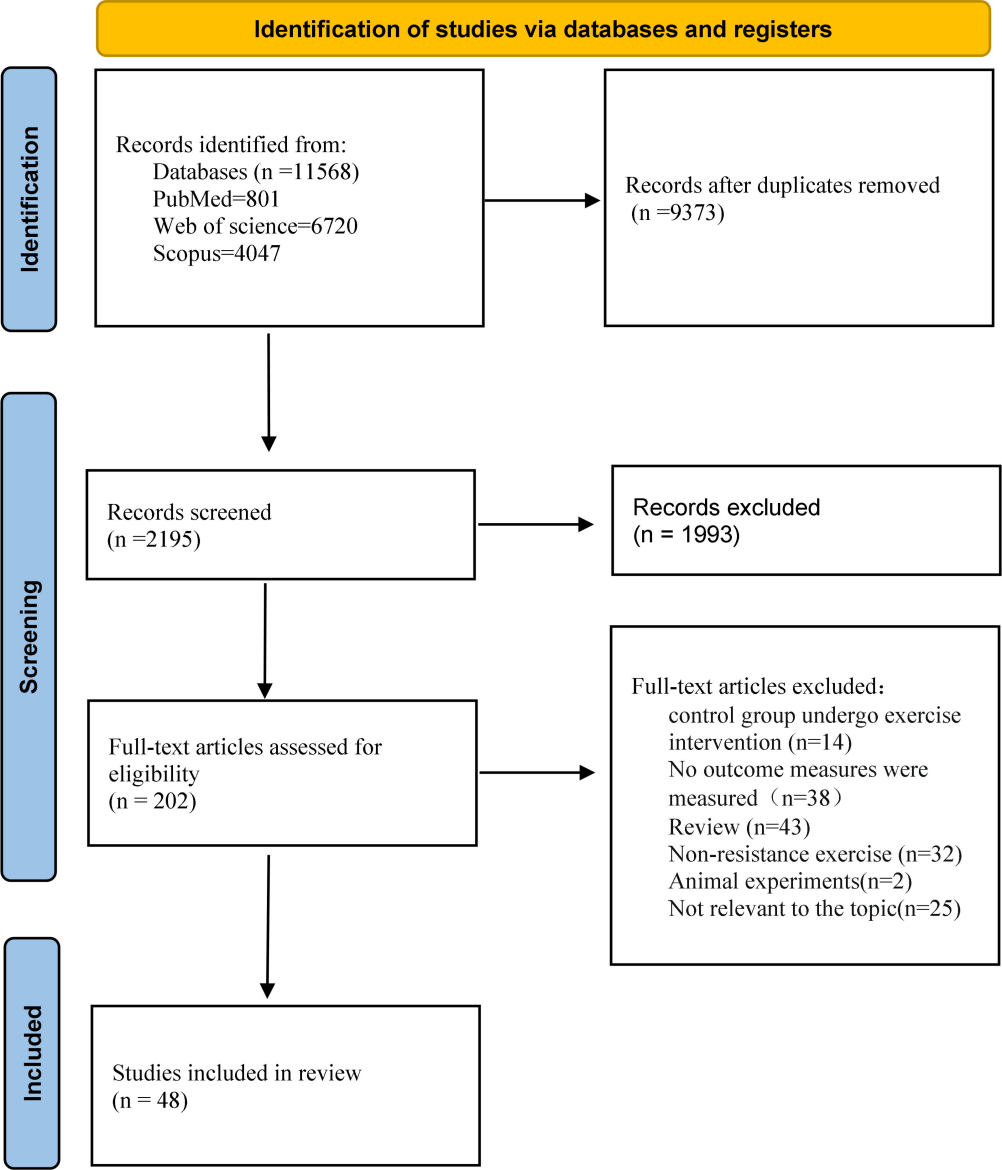
Flowchart of the selection of trials.

**Figure 2** shows the complete process of literature screening. A total of 7,064 articles were retrieved from the database. Following the removal of duplicate literature and the screening of article titles and abstracts, two authors independently assessed the full texts of the remaining 202 potential articles that might meet the inclusion criteria. Following the exclusion of studies that did not meet the inclusion criteria, 48 articles were included in the final study (25–31, 34–72) (see **Supplementary Table 2**)

### 3.2 Studies retrieved and characteristics

A total of 48 studies were conducted, involving 3,843 subjects from different countries and regions. Of the 48 studies, 13 were from the United States (25, 31, 34–36, 39, 54, 57–60, 62, 66), 9 were from Denmark (42, 47, 64, 65, 69, 70, 72–74), 5 were from China (26, 28, 48, 50, 63), 3 were from Australia (27, 29, 56), 3 were from Canada (44, 45, 71), 3 were from Germany (37, 40, 55), 2 were from Belgium (30, 43), 2 were from the Netherlands (51, 61), 2 were from Norway (46, 53), 2 were from Spain (38, 52), 1 was from Greece (68), 1 was from Korea (49), 1 was from Turkey (67), and 1 was from Brazil (41).

The studies included in this review encompass a wide range of cancer types, including breast, rectal, head and neck, prostate, lung, nasopharyngeal, and other cancers. A total of 21 studies were conducted on breast cancer (25, 31, 35–41, 57, 60, 66–68), followed by 6 studies on prostate cancer (27, 30, 46, 51, 56, 62), 4 studies on head and neck cancer (34, 47, 55, 59), 4 studies on gastrointestinal cancer (26, 29, 43, 63), 2 studies on lung cancer (44, 48), and 1 study on nasopharyngeal carcinoma (50). The remaining 10 studies were not limited to specific cancers (28, 45, 53, 58, 61, 69, 71–74).

The frequency, form, intensity, and duration of exercise interventions varied across the studies included in the systematic review. In most trials, two to three times per week was the usual frequency of exercise. Three main forms of exercise interventions were included, with 25 studies including resistance exercise interventions (26–28, 31, 35, 37, 39–41, 43, 49, 51, 55, 62, 66) and 11 studies combining resistance and aerobic interventions (25, 29, 34, 48, 52, 53, 56, 60, 67, 69, 71); the remaining 12 studies intervened with resistance exercise combined with other forms of exercise (including interval exercise, flexibility, relaxation, and massage) (36, 38, 42, 45, 57–59, 61, 68, 72–74). One study adopted high-intensity intermittent exercise combined with resistance as part of exercise intervention (61), two studies combined resistance, aerobic, and flexibility as part of exercise intervention (57, 68), and two studies included stretching and massage relaxation exercises (45, 73).

With regard to the report on the main results, 36 studies reported on physical fitness-related indicators, 40 studies reported on QOL-related indicators, and only 19 studies reported on fatigue-related outcomes. While the majority of studies included a common resistance exercise program, comprising chest press, leg press, chest extension, leg extension, knee extension, and leg curl, five studies did not mention a specific intervention exercise program (37, 43, 57, 68, 69). The duration of the studies ranged from 5 weeks to 52 weeks, but the majority of the studies had a duration of 12 weeks.

Upper and lower extremity muscle strength is the primary indicator of physical fitness when it comes to the measurement of research outcomes. The main testing instruments were the 1-repetition maximum test, the 8-repetition maximum test, and the 10-repetition maximum test. The results are expressed as the maximum weight that can be lifted in one repetition. Other metrics include VO_2_max, 6MWD, and others. The majority of the studies employed the EORTC QLQ-C30 as the QOL test tool, while others used the Functional Assessment of Cancer Therapy-General (FACT-G) and the 36-Item Short Form Survey (SF-36); the results were assessed by questionnaire scores. Tests of fatigue in patients with cancer include Functional Assessment of Cancer Therapy-Fatigue (FACT-F), Brief Fatigue Inventory (BFI), Multidimensional Fatigue Inventory (MFI), and Piper Fatigue Scale (PFS), and the results are also expressed in the form of questionnaire scores.

The characteristics of the included literature are shown in **Table 1**.

**Table 1:** Characteristics of studies included in the systematic review.

First author	Treatment state	Design	Population/Female patients (%)	Sample	Age (mean ± SD)	Intervention characteristics			Comparator	Measurement methods	Outcomes	Drop out
						Exercise program	Frequency/Intensity	Duration				
Resistance exercise												
(43), Belgium	Post-treatment, time not reported	RCT	Patient with rectal cancer (33.3)	T: 6 C: 6	T: 61.5 C: 64.5	NA	3 × 30–40 min/week 8–12 rep ×1–3/exercise	5 weeks	Usual care	FACIT-F; FACT-G	QOL Fatigue	T: 0 C:0
(26), China	>24–48 months, post-treatment	RCT	Patient with gastrointestinal cancer (44)	T: 94 C: 96	T: 55.4 ± 11.6 C: 52.3 ± 12.4	Leg extension, leg curl, leg press, shoulder internal and external rotation, seated row, latissimus pull down, shoulder flexion and extension	2 × 60 min/week 8–12 rep ×3/exercise at 60%–80% of 1RM	12 weeks	Relax control	1RM; EORTC QLQ-C30	Physical fitness QOL	T: 8 C: 6
(27), Australia	>12 months, post-treatment	RCT	Patient with prostate cancer (0)	T: 13 C: 12	T: 69.3 ± 2.3 C: 71.8 ± 1.8	Home-based progressive resistance training	3×/week 8–12 rep × 3/exercise	52 weeks	Usual care	Isometric dynamometer; SF-36	Physical fitness QOL	T: 3 C: 2
(31), Australia	>6 months, post-treatment	N-RCT	Breast cancer survivor (100)	T: 27 C: 27	64 ± 7	Chest press, leg press, leg extension, biceps curl, triceps press down, overhead press, seated row, leg curl, abdominal crunch, and lower back hyperextensions	2×/week 8–12 rep ×2/exercise at 52%–69% of 1RM	10 weeks	Baseline	1RM; SF-36	Physical fitness QOL	0
(28), China	Post-treatment, time not reported	RCT	Elderly patient with cancer (NA)	TC: 30 HIRT: 30 LIRT: 30 C: 30	≥55	Standing row, bench press, standing upper limbs dumbbell press, lying leg lifts, prone leg raises, and prone leg curls	3×/week 3 min × 3/exercise at 30%–60% of 1RM	12 weeks	Usual care	BFI; 1RM; QLQ-CCC	Physical fitness QOL Fatigue	HIRT: 5 LIRT: 3 C: 4
(39), USA	>12 months, post-treatment	N-RCT	Elderly breast cancer survivors (100)	T: 11	60 ± 2	Leg and chest press, knee extension, leg curl, row, abdominal crunch, and bicep curl	3 × (40–45 min)/week 15 rep × 3/exercise at moderate of 1RM	16 weeks	Baseline	PFS; SF-36; 1RM	Physical fitness QOL Fatigue	0
(35), USA	>24 months, post-treatment	RCT	Elderly breast cancer survivors (100)	T: 39 C: 38	70.9 ± 5.1	Chair stands, lunges, calf raises, one-arm row, chest press, and push-ups	3 × 60 min/week 10–15 rep × 2–3/exercise at 1RM	12 month	Baseline	LLFDI; SF-36	Physical fitness QOL	T: 7 C: 9
(41), Brazil	>6 months, post-treatment	RCT	Patient with breast cancer (100)	T: 12 C: 13	T: 55.0 (5.8) C: 54.3 (5.2)	Leg press, stiff-legged deadlift, barbell bench press, supinated lat pull down, and sit-ups	1×/week 8–12 rep × 3/exercise at 1RM	8 weeks	Usual care	10RM	Physical fitness	T: 1 C: 0
(55), Germany	Undergoing treatment	RCT	Patient with head and neck cancer (25)	T: 10 C: 10	T: 60.2 ± 4.7 C: 61.5 ± 15.7	Leg press, a latissimus pull-down, and a chest press	3 × 30 min/week 8–12 rep × 3/exercise	NA	Usual care	MFI; FAACT	QOL Fatigue	T: 0 C: 0
(62), USA	>3 months, post-treatment	RCT	Patient with prostate cancer (0)	RT+PRO: 16 PRO+STR: 19	T: 68.6 ± 8.4 C: 66.3 ± 9.0	Leg press, leg curl, leg extension, chest press, shoulder press, seated row, lat pulldown, plank, hip bridge, and dead bug	3 × 50 min/week 8–15 rep × 3/exercise at 60%–83% of 1RM	12 weeks	Stretch+Pro	10RM; FACT-G/FACT-P; BFI	Physical fitness QOL Fatigue	T: 3 C: 2
(51), Netherlands	>3 months, post-treatment	RCT	Patient with prostate cancer (0)	EXPRO: 30 EXPLA: 30 C: 36	EXPRO: 73 ± 7 EXPLA: 71 ± 7 C: 71 ± 7	Chest press with lateral pulldown and shoulder press with horizontal row, leg press, and leg extension	2–3×/week 5–8 rep × 3/exercise at 60%–70% of 1RM	20 weeks	Usual care	1RM	Physical fitness	EXPRO:13 EXPLA:11 C: 6
(66), USA	Undergoing treatment	RCT	Patient with breast cancer (100)	T: 10 C: 10	T: 57.5 ± 23.0 C: 56.6 ± 16.0	Lateral and frontal raises, horizontal chest press, lateral pulldown, alternating biceps curls with dumbbells, triceps extension, leg press, leg extension, leg curl, standing calf raises, and three different types of abdominal exercises	2 × 60 min/week 6–12 rep × 3/exercise	21 weeks	Usual care	1RM	Physical fitness	0
(49), Korea	>24 months, post-treatment	RCT	Patient with breast cancer (100)	T: 15 C: 15	T: 54.7 ± 5.1 C: 55.4 ± 4.3	Leg press seated row, leg extension, shoulder press, back extension, arm extension, hip adduction, and hip abduction	2–3 × 50 min/week 8–16 rep × 3–4/exercise at 40%–80% of 1RM	12 weeks	Usual care	Muscle strength measuring instrument	Physical fitness	NA
(37), Germany	Undergoing treatment	RCT	Patient with breast cancer (100)	T: 77 C: 78	T: 55.2 C: 56.4	NA	2×/week 8–12 rep × 3/exercise at 60%–80% of 1RM	12 weeks	Relax	EORTC QLQ-C30; FAQ	QOL Fatigue	T: 3 C: 2
(40), Germany	Undergoing treatment	RCT	Patient with breast cancer (100)	T: 49 N:46	T: 52.2 ± 9.9 C: 53.3 ± 10.2	EX comprised 8 different machine-based progressive resistance exercises	2 × 60 min/week 8–12 rep × 3/exercise at 60%–80% of 1RM	12 weeks	Relax	EORTC QLQ-C30/BR23; FAQ	QOL Fatigue	T: 3 C: 3
(30), Belgium	Undergoing treatment	RCT	Patient with prostate cancer (0)	T: 24 C: 24	T: 67.9 ± 7.1 C: 71.9 ± 8.1	Abdominal, pectoral, deltoid, trapezius, latissimus dorsi, erector spinae, biceps, triceps, quadriceps, hamstrings, gastrocnemius, soleus, and gluteus	3×/week 8–12 rep × 1–3/exercise	5–8 weeks	Usual care	FACIT-F; FACT-G	QOL Fatigue	T: 1 H: 3 C: 2
(44), Canada	Pre-treatment	N-RCT	Patient with lung cancer (58.8)	15	66.7 (50–85)	Leg press, chest press, seated row, leg extension, leg curl, shoulder press, lat pull down, and an abdominal exercise	3×/week 10–12 rep ×2–3/exercise at 60%–85% of 1RM	10 weeks	Baseline	1RM; SF-36/FACT-L; FACT-F	Physical fitness QOL Fatigue	2
(46), Norway	Undergoing treatment	RCT	Patient with prostate cancer (0)	T: 28 C: 30	T: 66 (54–76) C: 66 (54–76)	Smith machine half squat, leg press, Smith machine standing calf raises, knee flexion, knee extension, chest press, seated row, seated shoulder press, and biceps curl	3×/week 10 rep ×1–3/exercise at 40%–90% of 1RM	16 weeks	Usual care	1RM; EORTC QLQ-C30	Physical fitness QOL	T: 6 C: 3
(47), Denmark	>2 months, post-treatment	RCT	Patient with head and neck cancer (22)	T: 20 C: 21	T: 55 ± 7 C: 58 ± 7	Leg press, knee extension, hamstring curls, chest press, sit ups, back extensions, and lateral pull down	2×/week 8–15 rep × 2–3/exercise	12 weeks	Usual care	EORTC QLQ-C30	QOL	T: 1 C: 6
(50), China	Undergoing treatment	RCT	Nasopharyngeal carcinoma (35)	T: 67 C: 65	T: 44.7 ± 15.1 C: 46.2 ± 13.5	Leg extension, leg curl, leg press, shoulder internal and external rotation, seated row, latissimus pull down, shoulder flexion and extension, and butterfly and butterfly reverse	2 × 60 min/week	12 weeks	Relax	EORTC QLQ-C30	QOL Fatigue	T: 6 C: 8
(54), USA	Post-treatment, time not reported	RCT	Breast cancer survivors (100)	T: 20 C: 19	T: 51.2 8.5 C: 52.7 9.4	Leg extension, leg curl or Romanian deadlift, lat pull down, machine bench press, seated row, back extension, prone hold, or sit ups	3 × 60 min/week 8–10 rep × 3/exercise	16 weeks	Usual care	FACIT-F; FACT-G;1RM	Physical fitness QOL Fatigue	T: 1 C: 4
(63), China	Undergoing treatment	N-RCT	Patient with colorectal cancer (52.4)	42	57.9 ± 10.45	Lower limbs elastic-band resistance exercise	2 × 40 min/week	4.5 months	Baseline	1RM; EORTC QLQ-CIPN20/EORTC QLQ-C30	Physical fitness QOL	3
(64), Denmark	Undergoing treatment	RCT	Breast cancer survivors (100)	T: 75 C: 78	T: 51.5 ± 9.6 C: 52.0 ± 9.3	Chest press and latissimus pull down	3 × 60 min/week 5–12 rep × 2–3/exercise at 70%–90% of 1RM	12 weeks	Step counter	1RM; EORTC QLQ	Physical fitness QOL	T: 11 C: 11
(65), USA	No treatment	N-RCT	Breast cancer survivors (100)	YRT: 12 ORT: 8	NA	Chest press, back pulldown, shoulder press, biceps curl, triceps pushdown, leg press, leg extension, and leg curl	2×/week 8–12 rep × 3/exercise at 50%–80% of 1RM	8 weeks	Baseline	1RM; BIRS	Physical fitness QOL	YRT: 1 ORT: 1
(70), Denmark	~3 weeks, post-treatment	RCT	Patient with breast cancer (100)	T: 82 C: 76	T; 53 (33–73) C: 52 (30–74	Involved exercises for upper limb, lower limb, and core	2×/week	20 weeks	Usual care	QLQ C-30; FACIT-F	QOL Fatigue	T: 14 C: 14
Resistance combined with aerobic exercise																			
(29), Australia	Undergoing treatment	N-RCT	Patient with colorectal cancer (30)	10	54.6 ± 14.1	RT: chest press, seated row, lat pull down, leg press, leg extension, and leg curl AE: walking or jogging on a treadmill and cycling or rowing on a stationary ergometer	RT: 2 × 60 min/week; 6–12 rep × 2–4/exercise AET: 2 × 20 min/week; 60%–80% HR max	10 weeks	Baseline	1RM; EORTC QLQ C30; MFSI-SF	Physical fitness QOL Fatigue	1
(25), USA	1–36 months, post-treatment	RCT	Patient with breast cancer (100)	T: 47 C: 47	T: 45.91 ± 8.21 C: 51.87 ± 8.21	RT: chest press, leg press AE: dance, running, etc.	RT: 2 × (20–25 min)/week; 8–15 rep × 2/exercise, 10–20 BORG scale AET: 2 × (35–40 min)/week; 55–80 HRR	12 weeks	Usual care	Bruce protocol; 8RM; SF-36	Physical fitness QOL	T: 2 C: 3
(34), USA	Undergoing treatment	RCT	Patient with head and neck cancer (19)	T: 12 C: 14	57 ± 11	RT: wall push-ups, standing row, chest press, horizontal fly biceps curl, hip abduction, hip diagonal, leg press, heel raises, and wall squats AE: walking	RT: 2 × (20–60 min)/week at 5–20 lbs AE: 5×30 min/week; 75% HRmax	12 weeks	Usual care	MFI; dynamometer	Physical fitness Fatigue	Na
(60), USA	Within 6 months, post-treatment	RCT	Breast cancer survivor (100)	T: 50 C: 50		RT: leg press, chest press; lunge; seated row; leg extensions, triceps extensions; leg flexion, and biceps curl AE: treadmill walking/running, rowing machine, and stationary bicycle	RT: 3 × (50–80 min)/week 10–15 rep × 3/exercise at 60%–80% of 1RM AE: 3 × (30–50 min)/week at 65%–80%VO_2_max	16 weeks	Usual care	1RM; FACT-B/SF-36; BFI	Physical fitness QOL Fatigue	Na
(67), Turkey	Post-treatment, time not reported	N-RCT	Breast cancer survivor (100)	T: 24 C: 24	45.0 ± 2.2	RT: 10-min warm up, followed by 40-min leg and hip workout by using elastic band and ball, and 10-min cool down AE: walking and cycling	RT: 2 × 60 min/week AE: 3 × 50 min/week at 50%–60% HRmax	12 weeks	Usual care	EORTC-QLQ-C30;	QOL	0
(53), Norway	Within 24 months, post-treatment	RCT	Patient with gynecologic cancer (100)	T: 29 C: 31	56.9 ± 13.3	RT: using bodyweights, elastic bands, and dumbbells at moderate-to-high intensity AE: walked on the treadmill	RT: 2×25 min/week AE: 2×30 min/week	16 weeks	Usual care	1RM	Physical fitness	T: 6 C: 13
(48), China	>1 week post-treatment	RCT	Patient with lung cancer (36)	T: 26 C: 26	T: 56.04 ± 11.67 C: 58.03 ± 7.71	RT: involved resistance training and Baduanjin AE: Baduanjin	RT: 3–5×50 min/week at 30%–50% of 1RM AE: 3–5×50 min/week at 65%–75% of HRmax	8 weeks	Usual care	QLQ-C30/QLQ-LC13	QOL	T: 7 C: 8
(52), Spain	2–5 years, post-treatment	RCT	Breast cancer survivor (100)	T: 8 C: 8	T: 50 ± 5 C: 51 ± 10	RT: chest press, shoulder press, leg extension, leg curl, leg press, leg calf rise, abdominal crunch, low back extension, arm curl, arm extension, and lateral pull-down AE: cycle-ergometer	RT: 3 × 90 min/week 12–15 rep × 3/exercise AE: 3 × 30 min/week; 70%–80% HRmax	8 weeks	Usual care	EORTC QLQ-C30;	Physical fitness QOL	NA
(56), Australia	>2 months, undergoing treatment	RCT	Patient with prostate cancer (0)	T: 29 C;28	T: 69.5 ± 7.3 C: 70.1 ± 7.3	RT: chest press, seated row, shoulder press, triceps extension, leg press, leg extension, and leg curl, with abdominal crunches AE: cycling and walking/jogging	RT: 2×/week; 6–12 rep × 2–4/exercise at 70% of 1RM AE: 2 × (15–20 min)/week at 65%–80% HRmax	12 weeks	Usual care	1RM; SF-36/EORTC QLQ-C30	Physical fitness QOL	T: 1 C: 1
(69), Denmark	At least one cycle, undergoing treatment	RCT	Patient with cancer (75)	T: 106 C: 107	T: 47.1 (10.8) C: 47.8 (10.4)	RT: large muscle group AE: stationary bicycles	9 h/week RT: NA AE: 85%–95% HRmax	6 weeks	Usual care	Fact-An	QOL	T: 29 C: 27
(71), Canada	3–12 weeks, undergoing treatment	RCT	Elderly patients with cancer (80)	T: 10 C: 10	T: 67.5 C: 69.5	RT: upper limbs (single arm dumbbell row, push-ups, and Pallof press for dorsal, pectoral, and abdominal parts, respectively) and lower limbs (hip thrusts and goblet squat for quadriceps and hamstrings) AE: treadmill familiarization	RT: 2×/week; 10–15 rep/exercise AE: 3 × 50 min/week at 40%–75% HRmax	12 weeks	Relax+Str	FACIT-F; EORTC QLQ-C30/FACT-G	QOL Fatigue	T: 4 C: 1
Resistance combined with other exercises																			
(61), Netherlands	Post-treatment, time not reported	N-RCT	Cancer survivor (84)	T: 49 C: 22	T: 48 ± 8 C: 58 ± 11	RT: vertical row, leg press, bench press, pull over, abdominal crunch, and lunge IT: cycling two times	RT: 2×/1–12 weeks at 65%–80% of 1RM; 1×/13–18 weeks at 35%–40% of 1RM IT: 8 min consisted of alternating 30 s at 65% of the maximal short exercise capacity and 60 s at 30%.	18 weeks	Usual care	Indirect 1RM; MFI EORTC QLQ-C30	Physical fitness QOL Fatigue	T: 19 C: 0
(57), USA	Regardless of treatment/recovery phase	N-RCT	Breast cancer survivor (100)	C1: 21 C2: 31 (a year late)	C1: 59.2 ± 10.8 C2: 60.1 ± 10.2	RT: NA AE: treadmill, cycle ergometers, elliptical trainers, and recumbent stepping trainers Ba and Fl: balance ball exercises, ball and balloon tosses, reaches, etc.	2 × 90 min/week RT: 8–12 rep×1–2/exercise at 60%–70% 1RM AE: 70%–85% HRmax	12 weeks	Baseline	FACT-G; 1RM	Physical fitness QOL	10
(36), USA	>12 months, post-treatment	RCT	Elderly breast cancer survivor (100)	T: 52 C: 54	T: 62.3 (6.7) C: 62.2 (6.7)	Combination of dumbbells, barbells and weighted vests to apply resistance and focused on exercises that targeted the leg, hip, chest, and back and using movement patterns similar to those used in activities of daily living	2 × 30 min/week 8–12 rep × 1–3/exercise at 60%–80% of 1RM	12 months	Placebo	1RM; SF-36	Physical fitness QOL	T: 16 C: 23
(38), Spain	Within 10 years, post-treatment	RCT	Breast cancer survivor (100)	T: 32 C: 28	T: 52.6 (8.8) C: 52.0 (9.4)	RT: bilateral deadlift, bilateral seated row, bilateral squat, and bilateral seated bench press Homebased physical activity: undertaking ≥ 10,000 steps per day	2 × 60 min/week 12–24 rep × 2/exercise at 40%–70% of 1RM	12 weeks	Usual care	Dynamometer; FACT-F; FACT-B	Physical fitness QOL Fatigue	T: 2 C: 0
(72), Denmark	Undergoing treatment	N-RCT	Patient with cancer (56)	82	40	RT: leg press, a chest press, and a lat machine AE: interval training on a stationary bicycle Relax: progressive relaxation Awareness training: balance/coordination grounding and integration of the senses Massage	2–3×/week; RT: 5–8 rep × 3/exercise at 80%–95% of 1RM AE: 10 min at 60%–100% HRmax	6 weeks	Baseline	1RM; EORTC QLQ-C30	Physical fitness QOL	29%
(45), Canada	Undergoing treatment	N-RCT	Patient with cancer (80.8)	575	53.5 ± 10.8	RT: leg, chest, back, shoulder, arm, and abdominal muscle groups AE: NA Fle: emphasis on areas with range of motion limitations	2 × 60 min/week RT: 15–20 rep/exercise, RPE = 13 AE: 11–13 RPE	12 weeks	Baseline	SF-36	QOL	171
(73), Denmark	At least one cycle, post-treatment	RCT	Patient with cancer (73)	T: 135 C: 134	T: 47.2 (10.7) C: 47.2 (10.6)	RT: leg press, a chest press, a pull down, an abdominal crunch, a lower back, and a knee extension AE: stationary bicycles Low-intensity training comprised three components: relaxation, body awareness, and restorative training and massage	3 × 90 min/week; RT: 5–8 rep × 3/exercise at 70%–100% of 1RM AE: 85%–95% HRmax	6 weeks	Usual care	EORTC-QLQ-C3; SF-36; 1RM	Physical fitness Fatigue	T: 17 C: 17
(68), Greece	~3 years, post-treatment	N-RCT	Breast cancer survivor (100)	T: 13	58.31 ± 3.13	RT: legs, arms, shoulders, chest, back, and trunk AE: aerobic dance Relax exercises	2×/week RT: 10–12 rep × 4/exercises AE: 70%–80% HRmax	8 weeks	Baseline	EORTC QLQ-C30	QOL	NA
(74), Denmark	At least one cycle, undergoing treatment	N-RCT	Patient with cancer (61)	T: 23	40 (18–65)	RT: leg press, a chest press, and a lat machine AE: stationary bicycles Low-intensity training comprised three components: relaxation, body awareness, and restorative training and massage	3 × 90 min/week RT: 5–8 rep × 3/exercise at 85%–95% of 1RM AE: 60%–100% HRmax	6 weeks	Baseline	1RM; EORTC QLQ-C30; SF-36	Physical fitness QOL	4
(42), Denmark	At least one cycle, undergoing treatment	N-RCT	Patient with breast cancer (100)	70	42.8	RT: a leg press, a chest press, and a lat machine AE: stationary bicycles Relax: progressive relaxation Awareness training: Ba/co grounding and integration of the senses Massage	3 × 90 min/week RT: 5–8 rep × 3/exercise at 85%–95% of 1RM AE: 85%–95% HRmax Fle: 4×30 min/week	6 weeks	Baseline	1RM	Physical fitness	8
(58), USA	>36 months’ post-treatment	N-RCT	Cancer survivor (91.5)	59	59 ± 12	RT: lower body and upper body AE: treadmill walking, cycle ergometer, and elliptical trainer Ba and Relax: reaches, bends, balloon and ball toss exercises, etc.	2 × 90 min/week; RT: 8–12 rep × 1–2/exercise at 60%–70% of 1RM AE: 70%–85% HRmax	12 weeks	Baseline	1RM; FACT-G	Physical fitness QOL	17
(59), USA	Post-treatment, time not reported	N-RCT	Patients with head and neck cancer (50)	12	68	RT: pushing hands together or pulling them apart in front of the chest, pulling exercises for the back and shoulder with a rubber band, etc. Co: finger coordination, balance exercises Str/Relax: stretching of the neck, chest, progressive muscle relaxation	2 × 50 min/week RT: 10–15 rep × 1–3/exercise at 11–15 RPE	12 weeks	Baseline	EORTC QLQ-C30	QOL	2

RCT, randomized controlled trial; N-RCT, non-randomized controlled trial; T, test group; C, control group; TC, tai chi; HIRT, high-intensity resistance training; LIRT, low-intensity resistance training; RT, resistance training; PRO, protein; STR, stretching; EXPRO, exercise protein; EXPLA, exercise placebo; YRT, 40–59 years; ORT, 60–80 years; NA, no attention; AE, aerobic exercise; IT, interval training; Ba, balance; Fl, flexibility; Co, coordination.

FACIT-F, Functional Assessment of Chronic Illness Therapy-Fatigue; FACT-F, Functional Assessment of Cancer Therapy-Fatigue; FACT-G, Functional Assessment of Cancer Therapy-General; EORTC QLQ-C30, European Organization for Research and Treatment of Cancer QOL Questionnaire-C30; SF-36, Short-form-36; QLQ-CCC, QoL Questionnaire for Chinese Cancer Patients Receiving Chemobiotherapy; BFI, Brief Fatigue Inventory; PFS, Piper Fatigue Scale; LLFDI, Late-Life Function and Disability Instrument; MFI, Multidimensional Fatigue Inventory; FAACT, Functional Assessment of Anorexia/Cachexia Therapy; FACT-P, Functional Assessment of Cancer Therapy-Prostate; FAQ, Fatigue Assessment Questionnaire; FACT-L, Functional Assessment of Cancer Therapy-Lung; EORTC QLQ-CIPN20, European Organization for Research and Treatment of Cancer QOL Questionnaire-Chemotherapy-Induced Peripheral Neuropathy20; BIRS, Relationships Scale; MFSI-SF, Multidimensional Fatigue Symptom Inventory; FACT-B, Functional Assessment of Cancer Therapy-Breast; FACT-An, Functional Assessment of Cancer Therapy-Anemia Subscale.

### 3.3 Quality assessment

**Figure 3:**
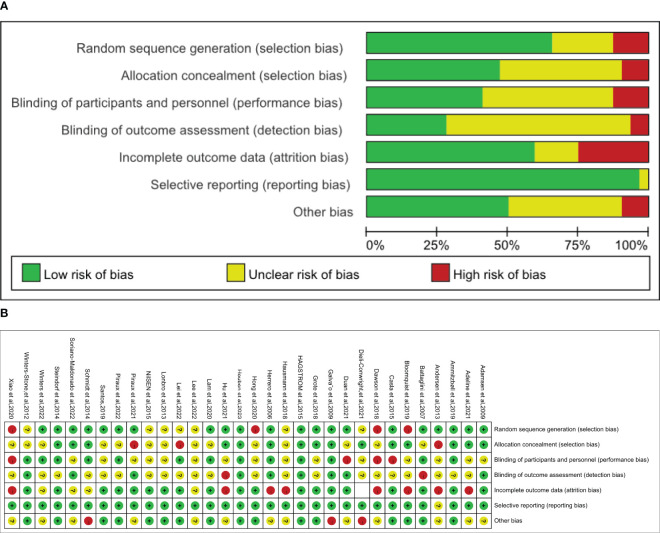
**(A)** Quality assessment of selected studies. **(B)** Risk of bias graph and summary.

A total of 48 studies were included in the analysis, comprising 32 RCTs and 16 non-randomized controlled intervention trials (N-RCTs). The results of the assessment of the quality of the RCT literature are presented in **Figure 3**. In summary, the risk of bias for the included studies was low. The majority of the risk of bias was attributable to issues related to blinding of study subjects or investigators, blinding of outcome measures, and incomplete outcome follow-up. Moreover, the majority of researchers provided comprehensive descriptions of the methods employed for the generation and distribution of random sequences. Because of the nature of the intervention and the subjectivity of outcome assessment instruments, the implementation and execution of blind methods become more challenging. The results of the quality assessment of the 16 experimental studies of N-RCTs are presented in **Table 2**. Only one of the studies had a control group, and the rest were self-controlled. Most of the studies scored above 12 points, except for two studies that scored low (below 11 points).

**Table 2:** MINORS score for non-RCTs.

Studies	Score
(29), Australia	12
(61), Netherlands	13
(31), USA	12
(39), USA	12
(57), USA	14
(72), Denmark	8
(67), Turkey	20
(45), Canada	14
(68), Greece	13
(74), Denmark	10
(42), Denmark	13
(44), Canada	14
(58), USA	14
(59), USA	14
(63), China	13
(65), Denmark	11

### 3.4 Outcomes

#### 3.4.1 Physical fitness

**Table 3** shows the changes in physical fitness after exercise interventions in patients with cancer in various research experiments, and 36 studies have investigated the effect of resistance on physical fitness in patients with cancer. There are many evaluation indexes of physical fitness, including but not limited to muscle strength, VO_2_max, and 6MWD, among others. Muscle strength, such as leg pressure and chest pressure, is the primary evaluation indicator of physical fitness.

**Table 3:** Effect of resistance exercise on physical fitness of patients with cancer.

Study	Status	Measurement methods	Descriptive	Main reported outcomes
Resistance exercise				
(26), China	>24–48 months, post-treatment	1RM test	The maximum weight that can be lifted at one time (70%)	Significant between-group difference for leg press (*p* = 0.021) and leg extension (*p* = 0.041).
(27), Australia	>12 months, post-treatment	Isometric dynamometer; VO_2_max	Triceps brachii extension and knee extension, the best score was taken 3 times; maximum oxygen consumption	Muscle strength between the groups did not change significantly (*p* = 0.35), and lower limb muscle strength decreased significantly (*p* = 0.02). Physical function (balance and coordination) and VO_2_max there was no significant between the groups (*p* = 0.35), but the physical activity increased significantly (*p* = 0.05).
(31), Australia	>6 months, post-treatment	1RM test	Assessed with chest compressions and leg extension exercises	Show steady strength improvement. Significant between-group difference for chest press (*p* ≤ 0.05), leg extension (*p* ≤ 0.05). There were significant improvements from baseline to 6 months in upper limb strength, lower limb strength, balance coordination, endurance, and total function.
(28), China	Post-treatment, time not reported	1RM test	Test movements were completed at the established intensity and the last 1RM was recorded	The muscle strength of the control group did not decrease significantly, while the muscle strength of the TC and RT groups increased significantly (*p* < 0.05). Compared with the control group, the muscle strength of the RT group increased (*p* < 0.05).
(39), USA	>12 months, post-treatment	1RM; 6MWT	The maximum weight that can be lifted at one time; the distance walked quickly on a flat	Muscle strength increased by 25%–30% (*p* < 0.01), a significant between-group difference for chest press, knee extension, and leg press, 6-min walking speed increased by 4%, and body composition remained unchanged after intervention.
(35), USA	>24 months, post-treatment	1RM test	The maximum weight that can be lifted at one time	Compared with the flex group, the upper body muscle strength of the RT group increased more (2.5 kg, *p* = 0.048). Compared with the AE group, lower limb strength in the RT group increased more (8.2 vs. 2.7, *p* = 0.037).
(41), Brazil	>6 months, post-treatment	10RM test	10 maximum repetitions of leg push and bench press to test muscle strength	Leg (*p* < 0.02) and bench press (*p* < 0.01) of the RT group were significantly improved. The muscle strength ES was larger, and the bench press 10RM = 1.15. There was no difference at baseline.
(62), USA	>3 months, post-treatment	10RM test	10 maximum repetitions of leg push and bench press to test muscle strength	Significant for leg (*p* < 0.01), chest (*p* = 0.048), and the strength of leg push and bench press increased significantly compared with the non-exercise group.
(51), Netherlands	>3 months, post-treatment	1RM test	The maximum weight that can be lifted at one time	There was a significant difference in leg muscle strength between the two control groups over time (*p* < 0.01).
(66), USA	Undergoing treatment	1RM test	The maximum weight that can be lifted at one time	The strength change was statistically significant (*p* = 0.025), and the body fat decreased significantly (*p* = 0.004).
(49), Korea	>24 months, post-treatment	1RM test; sit-to-stand test	The maximum weight that can be lifted at one time; successively rise from a chair and sit down	Grip strength (*p* = 0.001), back strength (*p* = 0.014), sit-ups (*p* = 0.028), reaction time (*p* = 0.043), one leg standing with eyes closed (*p* = 0.028), and y-balance test comprehensive score (*p* = 0.022) had interaction, while flat support (*p* = 0.167) and sitting extension (*p* = 0.163) had no interaction.
(44), Canada	Pre-treatment	1RM test	The maximum weight that can be lifted at one time	The strength of chest push and bench press increased significantly by 32 kg (52%) and 15 kg (42%). In terms of objective physical function, 6MWD and chair standing times were significantly improved (*p* < 0.001).
(46), Norway	Undergoing treatment	1RM test	Maximum weight of bench press and leg push lift	Statistically significant for leg press and chest press (*p* < 0.001).
(54), USA	Post-treatment, time not reported	1RM test	The maximum weight that can be lifted at one time	Compared with the control group, the lower body strength increased significantly (*p* < 0.001).
(63), China	Undergoing treatment	1RM test; 6MWT	Take the average of the three maximum weights that can be lifted by leg push; the distance walked quickly on a flat	Compared with baseline, 1RM was significantly improved at the last measurement (*p* < 0.001), 6MWD (endurance) distance improved by 6.4%.
(64), Denmark	Undergoing treatment	1RM test	The maximum weight that can be lifted at one time	The upper limb strength of the exercise group increased significantly (*p* < 0.05) (chest).
(65), USA	No treatment	1RM test; BMI	The maximum weight that can be lifted at one time; body mass index	The muscle strength of the two groups was significantly improved after intervention, the upper limb strength of 10RM in the young group was increased by 80% and that of the old group was increased by 99% (*p* < 0.001), and the lower limb strength of 1RM in both groups was improved by 34% (*p* < 0.001), but the weight, BMI and waist circumference of the two groups were not improved.
Resistance combined with aerobic exercise				
(29), Australia	Undergoing treatment	1RM test; 6MWT	The maximum weight that can be lifted at one time (kg); the distance walked quickly on a flat	Leg press (*p* = 0.030) and leg extension (*p* = 0.046) raise 27.2% and 22.7%, respectively; there was a significant improvement (*p* < 0.05), 6MWD (6.9%) and backward walk (15.5%); reduction (*p* = 0.006) in heart rate of 10 beats per minute immediately after the completion of the test; fat loss.
(25), USA	1–36 months, post-treatment	8RM test; VO_2_max	Eight maximum repetitions and an estimated one maximum repetition; maximum oxygen consumption	Significant between-group differences for maximal strength chest, resistance strength chest resistance strength legs, maximal strength chest/weight, maximal strength legs/weight (*p* < 0.001), and VO_2_max increased significantly and maintained the effect after 6 months. Ex group significantly reduced the percentage of fat and increased lean body weight (*p* < 0.01), but the effect disappeared after 6 months, and there was no difference between the groups.
(34), USA	Undergoing treatment	Dynamometer; 6MWT	Measure the weight of each hand and the average weight; the distance walked quickly on a flat	Compared with the control group, the grip strength decreased slightly (*p* = 0.05), and the 6-min walking distance increased (*p* = 0.04).
(60), USA	Within 6 months, post-treatment	1RM test; VO_2_max	The maximum weight that can be lifted at one time; maximum oxygen consumption	Significantly in the chest press (*p* < 0.01), nationality adjusted the effect of exercise on maximal oxygen uptake. Spain also had a better effect than non-Spaniards, showing the adjustment effect in addition to race.
(53), Norway	Within 24 months, post-treatment	1RM test; VO_2_max	The maximum weight that can be lifted at one time; maximum oxygen consumption	The muscle strength of patients with cancer was significantly improved, and the peak value of VO_2_max was significantly increased (*p* = 0.009).
(52), Spain	2–5 years, post-treatment	Bench and leg-press machine; VO_2_max	Complete the movement until muscle fatigue; maximum oxygen consumption	There was a significant difference between the two groups (*p* < 0.05), and chest push (0.08) had a significant effect on total muscle mass, body fat, and cardiopulmonary function.
(56),Australia	>2 months undergoing treatment	1RM test; 6MWT	The maximum weight that can be lifted at one time; the distance walked quickly on a flat	The leg press force was significantly improved (*p* < 0.001), the chest press force was marginalized (*p* = 0.18), and the lean weight of patients (*p* = 0.047), 6MWD (*p* = 0.024), and 6-min backward walking distance (*p* = 0.039) were significantly improved.
(71), Canada	3–12 weeks, undergoing treatment	6WMT; BMI	The distance walked quickly on a flat; body mass index	There was a significant difference in the 6MWD. Grip strength increased (*p* = 0.04), lean weight increased (*p* = 0.02), body fat remained unchanged, and there was no difference in physical activity and energy intake.
Resistance combined with other exercises				
(61), Netherlands	Post-treatment, time not reported	Indirect 1RM test	The maximum weight that can be lifted at one time (65%–80%)	Vertical row (*p* < 0.01), leg press (*p* < 0.01), bench press (*p* < 0.01), pull over (*p* < 0.01), lunge (*p* < 0.01), abdominal crunch (*p* < 0.01), the fact that there were no significant differences in cardiopulmonary function between week 18 and week 68.
(57), USA	Regardless of treatment/recovery phase	1RM test; 6WMT	The maximum weight that can be lifted at one time; the distance walked quickly on a flat	After the intervention, the upper and lower limb muscle strength and 6MWD in the early and late start groups were significantly increased compared with baseline, but there was no significant difference between the two groups (*p* > 0.05).
(36), USA	>12 months, post-treatment	1RM test	The maximum weight that can be lifted at one time	The improvement of bench press (*p* < 0.01) and leg push (*p* < 0.03) was significantly greater than that of the control group. Walking speed improved significantly at 6 months (*p* < 0.04), and decreased in December, but still higher than the baseline.
(38), Spain	Within 10 years, post-treatment	Dynamometer; VO_2_max	Instrument measurement value; maximum oxygen consumption	The mean changes (SE) in the standardized full-body muscular strength index from baseline to week 12 were 0.335 (0.122) in the RTG and −0.383 (0.130) in the CG, *p* < 0.001, no effect on VO_2_max.
(72), Denmark	Undergoing treatment	1RM test; VO_2_max	The maximum weight that can be lifted at one time; maximum oxygen consumption	Muscle strength was significantly improved (*p* < 0.01), VO_2_max was improved by 16% on average, and the level of physical activity was increased (*p* < 0.05).
(73), Denmark	At least one cycle, post-treatment	1RM test; VO_2_max	The maximum weight that can be lifted at one time; maximum oxygen consumption	Compared with the control group, VO_2_max in the intervention group increased by an average of 10.7% (*p* < 0.001).
(68), Greece	~3 years, post-treatment	6MWT; BMI	Length of walking distance; body mass index	6MWD was significantly increased (*p* < 0.001), and lower limb muscle strength was improved. Compared with baseline, training reduced BMI and waist circumference (*p* < 0.001).
(74), Denmark	At least one cycle, undergoing treatment	1RM test; VO_2_max	The maximum weight that can be lifted at one time; maximum oxygen consumption	Leg press improved by 44% (*p* < 0.001), the chest press improved by 19% (*p* < 0.001), and the total strength increased by 32.5%, the average improvement rate of VO_2_max was 16%.
(42), Denmark	At least one cycle, undergoing treatment	1RM test; VO_2_max	The maximum weight that can be lifted at one time; body mass index	The chest pressure was improved by 39% (*p* < 0.001), and the leg pressure was improved by 44%. The average improvement rate of VO_2_max was 14.5% (*p* < 0.001), and the average weight gain was 1% (*p* < 0.009).
(58), USA	>36 months, post-treatment	1RM test; 6MWT	The maximum weight that can be lifted at one time; length of walking distance	The leg press and chest press were significantly changed (*p* < 0.001), and the 6MWD was significantly improved by 15.5% (*p* < 0.001).
(59), USA	Post-treatment, time not reported	6MWT	Length of walking distance	The average distance of 6MWD increased by 43.3 m (*p* = 0.01), and the head rotation increased by 11.2° (*p* = 0.042).

FACIT-F, Functional Assessment of Chronic Illness Therapy-Fatigue; FACT-F, Functional Assessment of Cancer Therapy-Fatigue; BFI, Brief Fatigue Inventory; PFS, Piper Fatigue Scale; MFI, Multidimensional Fatigue Inventory; FAQ, Fatigue Assessment Questionnaire; MFSI-SF, Multidimensional Fatigue Symptom Inventory; FACT-An, Functional Assessment of Cancer Therapy-Anemia Subscale; EORTC QLQ-C30, European Organization for Research and Treatment of Cancer Quality of Life Questionnaire-C30.

Seventeen studies reported the effect of resistance exercise intervention on physical fitness in patients with cancer, 16 studies demonstrated positive intervention effects of resistance exercise in patients with cancer, while 1 study reported reduced muscle strength but increased physical activity levels (27). Of these 16 studies, 8 reported significant improvements in leg press and chest press in patients with cancer compared to controls after exercise interventions (26, 31, 39, 41, 44, 46, 62, 64). Simonavice et al. (31) evaluated the effect of 6-month resistance training on physical fitness and QOL of patients with breast cancer. The results demonstrated a consistent and progressive enhancement in patient strength. Furthermore, significant improvements in balance, coordination, endurance, and overall function were observed in the study participants from baseline to month 6. Serra et al. (39) investigated the effects of 16 weeks of progressive resistance training on inflammation, fatigue, and physical functioning in patients with breast cancer under the supervision of an exercise physiologist. The results showed that resistance training not only improved muscle strength, but also resulted in a 4% increase in 6-min walking speed. The study found no significant changes in body composition compared to the control group. Peddle-McIntyre et al. (44) reported the feasibility and preliminary efficacy of progressive resistance exercise training (PRET) intervention for lung cancer survivors. The results showed significant therapeutic effects in objective physical, 6MWD, and chair standing. The results of the remaining eight studies (28, 35, 49, 51, 54, 63, 65, 66) not only included significant improvements in physical fitness, but Cheng et al. (28) also explored the effects of different intensities of tai chi (TC) and resistance exercise on fatigue and QOL in middle-aged and elderly Chinese patients with cancer. The results demonstrated that resistance exercise was more effective than TC in enhancing muscle strength, but TC was more efficacious than resistance exercise in improving sleep quality and mental health. In a study by Winters et al. (35), resistance was compared to the relaxation and aerobic exercise groups, respectively. There was a greater increase in upper limb muscle strength compared to the relaxation group, and a greater increase in limb muscle strength compared to the aerobic group; the lower limb strength increased more. Lee et al. (49) compared the effects of improvements in various aspects of physical fitness, with significant results being achieved in grip strength, back strength, sit-ups, reaction time, one-legged closed-eye stand, and Y-balance. Benton et al. (65) found that the younger group (40–59 years old) showed better improvement in upper limb strength than the older group (60–80 years old) and that resistance had no effect on body weight, BMI, or waist circumference. Moreover, of all the studies that have investigated the effects of resistance exercise on physical fitness in patients with cancer, a total of 11 studies have been conducted in which patients have participated in exercise tests following the completion of chemotherapy or radiotherapy; of these studies, 4 studies focused on breast cancer, 3 focused on prostate cancer, 1 focused on gastrointestinal cancer, and 4 focused on early-stage cancer. The results demonstrated that the resistance exercise intervention led to a favorable improvement in the study participants who had completed their cancer treatment. Most physical fitness indicators in patients with all types of cancer who completed treatment, including chest press and leg extension, exhibited statistically significant results. In addition, five studies had subjects who were still undergoing cancer treatment at the time of the exercise intervention: two studies on lung cancer, one on prostate cancer, one on lung cancer, and one on colorectal cancer. The findings of the study indicate that resistance exercise is an efficacious intervention for enhancing physical fitness in individuals undergoing cancer treatment. Nevertheless, a study was conducted on patients with breast cancer who underwent the exercise intervention without any additional treatment (65). However, it was demonstrated that, despite the absence of any therapeutic intervention, the implementation of an exercise regimen would yield comparable statistical benefits in terms of enhanced physical fitness.

Eight studies reported the effect of resistance exercise combined with aerobic exercise on physical fitness in patients with cancer. In comparison to the control group, most studies reported that resistance exercise combined with aerobic exercise had a positive effect on the physical fitness of patients with cancer (29, 52, 53, 56). In addition to the improvement of muscle strength, the studies also demonstrated significant effects, including the improvement of VO_2_max and 6MWD, the increase of muscle mass, the increase of lean weight, and the decrease of body fat rate. In the other four studies, Xiao et al. (34) reported the effect of resistance exercise combined with aerobic exercise intervention on patients with head and neck cancer undergoing radiotherapy. After the intervention, there was a slight decrease in grip strength but an increase in 6-min walk distance compared to the control group. Casla et al. (25) assessed the effect of aerobic and resistance exercise intervention on cardiorespiratory fitness in patients with early-stage breast cancer. The results showed significant increases in maximal oxygen capacity and muscle strength, which were also evident after 6 months of follow-up. Although there was an increase in lean body mass and a decrease in percent fat at the end of the intervention, the effects disappeared after follow-up. Dieli-Conwright et al. (60) showed that Hispanics had increased muscle strength and better VO_2_max improvement and reported the race-modifying role of exercise interventions in cancer treatment. Adeline et al. (71) explored the feasibility of combination of aerobic exercise and resistance exercise on the physical ability of elderly patients with cancer. The results showed that resistance exercise and aerobic exercise had significant effects on grip strength, 6-min walking distance, and lean weight of elderly patients with cancer, but had no effect on physical activity and energy intake. In these studies, subjects in four studies completed treatment related to cancer, and subjects in the other four studies were undergoing treatment for cancer. The studies that completed treatment included three on breast cancer and one on gynecologic cancer. Those undergoing treatment included one study on head and neck cancer, one on prostate cancer, one on rectal cancer, and one on early-stage cancer. The results demonstrated a significant impact on the enhancement of physical fitness through the integration of resistance exercise with aerobic training, both during the course of cancer treatment and across various types of patients with cancer. Statistically significant differences were observed between groups.

Eleven studies reported the effects of resistance exercise combined with other exercises on the physical fitness in patients with cancer. Four studies reported the significant effect of an exercise intervention on muscle strength and walking distance in patients with cancer (36, 57, 58, 68); Winters-Stone et al. (36) reported that although there was a decrease in 6MWD at 12 months, it was still higher than baseline values. Andrioti et al. (68) explored the effect of home-based tele-exercise training intervention on physical and mental health indicators in breast cancer survivors. In addition to the increase in lower limb muscle strength and 6MWD, they also found that exercise could reduce the BMI and waist circumference of patients. Six studies reported the effect of resistance exercise combined with other exercises interventions on muscle strength and VO_2_max of patients with cancer, with only two studies reporting no effect on VO_2_max (38, 61) and the other four studies reporting significant improvements in muscle strength and VO_2_max (42, 72–74). Felser et al. (59) explored the feasibility and impact of low and moderate-intensity exercise intervention on physical fitness and QOL in patients with head and neck cancer. They used 6MWD and head rotation as primary outcome indicators of physical function. The results showed significant changes in both. During the cancer treatment phase, subjects in seven studies participated in exercise interventions after completing the relevant treatment, and subjects in three studies were enrolled in an exercise testing program during cancer treatment and had completed at least one treatment cycle. Three studies were focused on breast cancer, one focused on head and neck cancer, and the remaining six studies did not target a specific cancer. The results of the 10 studies on the effects of resistance training combined with other exercise interventions on the physical functioning of patients with cancer demonstrated that the majority of the indices related to muscle fitness exhibited a significant improvement following the exercise interventions in comparison to the control group. However, only one study indicated that there was no significant change in VO_2_max following the exercise interventions. Soriano−Maldonado et al. (38) assessed the effects of 12-week supervised resistance training combined with home-based physical activity on physical fitness, cancer-related fatigue, depressive symptoms, QOL, and life satisfaction in female breast cancer survivors. The results of the study indicated that the exercise intervention significantly enhanced muscle strength in the upper and lower extremities, as well as the entire body, in patients with cancer. However, the patients reported no significant improvement in other health indicators, such as VO_2_max. Nevertheless, a study on breast cancer included subjects who had completed cancer treatment as well as those in the treatment stage. In 2018, Foley et al. (57). conducted a study investigating the effects of a 12-week community-based multimodal exercise program on physical function in breast cancer survivors. The results show that regardless of start delay, meaningful improvements in physical fitness were found after completing the community-based multimodal exercise program, but there was no significant difference between the two groups.

#### 3.4.2 Quality of life

**Table 4** shows the changes in QOL of patients with cancer after resistance exercise interventions across studies. A total of 40 studies reported the effect of resistance exercise interventions on the QOL of patients with various types of cancer.

**Table 4:** Effect of resistance exercise intervention on the QOL of patients with cancer.

Study	Status		Measurement methods	Descriptive	Main reported outcomes
Resistance exercise					
(43), Belgium	Post-treatment, time not reported		FACT-G	27 items, score 0–102; the higher the score, the better the QOL	No significant between-group difference (*p* > 0.05).
(26), China	>24–48 months, post-treatment		EORTC QLQ-C30	30 items, 0–100 points for each item, and more than 10 points are considered meaningful	Significant between-group difference for physical function (*p* = 0.035), role function (*p* = 0.041), social function (*p* = 0.047), appetite loss (*p* = 0.012), and fatigue (*p* = 0.024).
(27), Australia	>12 months, post-treatment		SF-36	Questionnaire survey evaluation	Significant between-group difference for vitality (*p* = 0.02), mental health (*p* = 0.04), and mental component summary (*p* = 0.02). The QOL has improved significantly.
(31), Australia	>6 months, post-treatment		SF-36	Questionnaire score evaluation, the score range is 0–100; the higher the better	The survey showed that the psychological and physical QOL did not change significantly at baseline, March, and June (*p* > 0.05).
(28), China	Post-treatment, time not reported		QLQ-CCC	35 items, 4 scales: physical, psychological, social, and general feeling; the higher the score, the better the state	Compared with the control group, the QOL in each intervention group was significantly improved after the intervention (*p* < 0.05).
(39), USA	>12 months, post-treatment		SF-36	The higher the score on the questionnaire, the better the QOL	QOL improved by 10% (*p* = 0.04), PCS (physical health) score improved by 8%, but MCS (mental) score remained unchanged (54 ± 3 vs. 55 ± 3; *p* = 0.93).
(35), USA	>24 months, post-treatment		SF-36	The scale score is 0–100. The higher the score, the better the function	Compared with flex exercise, the improvement of physical function in the SF-36 scale was more obvious (*p* = 0.066).
(55), Germany	Undergoing treatment		FACT	Scores of five questionnaires on physical, social, family, and emotional functions	There was no significant difference in the QOL between the control group and the intervention group (*p* = 0.891).
(62), USA	>3 months, post-treatment		FACT-G FACT-P	The higher the score, the better the quality	The exercise group observed more significant improvement in FACT-G (*p* = 0.048) and specific prostate cancer QOL (*p* = 0.04). Any QOL indicator was statistically significant (*p* < 0.05).
(37), Germany	Undergoing treatment		EORTC QLQ-C30	Questionnaire score	The overall QOL in the ex-group increased but there was no significant difference (*p* = 0.37, ES = 0.15). Ex was significantly higher in role function (*p* = 0.035, ES = 0.31) and in pain (*p* = 0.040, ES = 0.25).
(40),Germany	Undergoing treatment		EORTC QLQ-C30/BR23	The questionnaire score is 0–100. The higher the function score, the better the quality. The higher the symptom score, the worse the quality	There were significant differences in physical function (*p* = 0.087), role function (*p* = 0.035), and social function (*p* = 0.046) between the intervention group and the control group, which reflected the effect of improving the QOL.
(30), Belgium	Undergoing radiation therapy		FACT-G	The higher the score, the better the status	There was no difference in QOL between the two groups (*p* = 0.414).
(44), Canada	Pre-treatment		SF-36/FACT-L	The higher the score, the better the status	SF-36: role physical (*p* = 0.072), body pain (*p* = 0.101), and physical health component (*p* = 0.092) showed marginal improvement, while others remained unchanged. There was no significant change in specific QOL before and after intervention (0.507).
(46), Norway	Undergoing treatment		EORTC QLQ-C30	The higher the questionnaire score, the better the QOL	No significant changes in QOL were observed (*p* > 0.05).
(47), Denmark	>2 months, post-treatment		EORTC QLQ-C30	The higher the questionnaire score, the better the QOL	Significant in global health (*p* < 0.001), physical function (*p* < 0.001), role function (*p* < 0.05), and fatigue (*p* < 0.05).
(50), China	Undergoing treatment		EORTC QLQ-C30	5 function scales, 0–100 points; the higher the score, the better the quality	Significantly higher scores were observed in the resistance exercise group, in terms of global QOL (*p* = 0.042), physical function (*p* = 0.031), social function (*p* = 0.046), and role function (*p* = 0.022).
(54), USA	Post-treatment, time not reported		FACT-G	0–108 points; the higher the score, the higher the QOL	The QOL was significantly improved (*p* = 0.015, ES = 0.16).
(63), China	Undergoing treatment		EORTC QLQ-CIPN20/EORTC QLQ-C30	The higher the symptom score, the more serious it is. The higher the overall score, the better the quality	QLQ-C30 had no significant change during the whole course of chemotherapy or exercise intervention (*p* = 0.556). For the symptom scale, the score gradually decreased over time, which had a significant effect on the reduction of cancer-specific symptoms (*p* = 0.039).
(64), Denmark	Undergoing treatment		EORTC QLQ (BR23	Score of functional scale and symptom scale	There was no significant difference between groups in the score of QOL scale (*p* > 0.05).
(65), USA	No treatment		BIRS	The higher the score, the more serious the symptom	After 8 weeks of training, the total score of BIRS in the young group and the old group decreased by 29% and 5%, and the effect on the young group was greater (*p* = 0.002). Both the young group and the old group reported significant improvement in strength and health (37% and 6%) and social disorders (32% and 17%).
(70), Denmark	~3 weeks, post-treatment		QLQ C-30 version 3	The higher the score, the better the QOL	The intervention had a significant impact on emotion (*p* = 0.02) and social function (*p* = 0.04). The intervention effect on other indicators of the scale did not reach statistical significance, but the results tended to the intervention group.
Resistance combined with aerobic exercise					
(29), Australia	Undergoing treatment		QOL	Questionnaire score	Except for emotional function, diarrhea, financial difficulties (*p* < 0.05), and less constipation (*p* = 0.078), the overall QOL has not changed much.
(25), USA	1–36 months, post-treatment		SF-36	36 items; the higher the score, the better the health status	Significantly higher SF-36 scores in mental and physical dimensions (*p* = 0.002 and *p* = 0.001, respectively) and significant improvements in all SF-36 subdomains, compared with CON, except for the role limitation due to emotional health.
(60), USA	Within 6 months, post-treatment		SF-36	Questionnaire score	The QOL of the two races has improved significantly, which is embodied in SWB, EWB, and FWB.
(67), Turkey	Post-treatment, time not reported		EORTC-QLQ-C30	The higher the score, the better the effect	Compared with the control group, there were significant differences in physical function (*p* = 0.001), social function (*p* = 0.009), and QOL (*p* = 0.001). Compared with the exercise group, the control group had significant differences in symptoms of nausea (*p* = 0.038), sleep disorder (*p* = 0.033), loss of appetite (*p* = 0.001), and financial impact (*p* = 0.004), and the degree of depression of patients was significantly reduced (*p* < 0.001).
(48), China	>1 week, post-treatment		QLQ-C30/QLQ-LC13	The higher the score on the functional scale, the better the functional status. The higher the score on the symptom scale, the worse the symptoms	Significant differences in physical function, role function, emotional function (*p* < 0.01), and social function (*p* < 0.05). On the symptom scale, fatigue, pain, dyspnea, sleep anxiety, and other aspects in the intervention group were significantly improved (*p* < 0.05).
(52), Spain	2–5 years, post-treatment		EORTC QLQ-C30	5 function scales, 0–100 points; the higher the score, the better the quality	The QOL was significantly improved (*p* = 0.002), and physical function (*p* = 0.04), VO_2_max, and peak power in the experimental group had a significant interaction (*p* < 0.05).
(56),Australia	>2 months, undergoing treatment		SF-36/QLQ-C30	SF-36 for general QOL and QLQ-C30 for cancer-specific QOL	The change scores in general health (*p* = 0.022), vitality (*p* = 0.019), and physical health comprehensive score (*p* = 0.020) were higher. The change in general health status was correlated with the change in body lean mass (*p* = 0.039), and the change in average muscle strength was close to significant (*p* = 0.064). QLQ-C30 found better change scores in cognition (*p* = 0.007), fatigue (*p* = 0.021), nausea (*p* = 0.025), and dyspnea (*p* = 0.017), and there were marginal differences in physical (*p* = 0.062), emotional (*p* = 0.098), pain (*p* = 0.092), and insomnia (*p* = 0.055).
(69), Denmark	At least one cycle, undergoing treatment		FACT-G	The higher the score, the better the QOL	FACT-G score (*p* = 0.21) or individual wellbeing score had no statistically significant effect; physical health (PWB) (*p* = 0.13), emotional health (EWB) (*p* = 0.87), social health (SWB) (*p* = 0.83), and functional health (FWB) (*p* = 0.26).
(71), Canada	3–12 weeks, undergoing treatment		FACT-G/EORTC QLQ-C30	The higher the FACT-G score, the better the quality. The higher the QLQ function score, the better the quality. The higher the symptom score, the worse the quality	EORTC QLQ-C30 global score increased in the MIX group only (*p* = 0.05). This improvement may be explained by trends towards increased global health status (*p* = 0.06), physical functioning (*p* = 0.06), and decreased fatigue (*p* = 0.09) in the MIX group. There was no difference in the total score of FACT-G between groups (*p* ≥ 0.12).
Resistance combined with other exercises					
(61),Netherlands		Post-treatment, time not reported	EORTC QLQ-C30	Questionnaire scores of 30 items	Physical functioning, role functioning, emotional functioning, cognitive functioning, social functioning, and fatigue (*p* > 0.05).
(57), USA		Regardless of treatment/recovery phase	FACT-G	Questionnaire score	Statistically significant (*p* < 0.05) differences in pre- and post-measurements for PWB, EWB, FWB, and TWB.
(36), USA		>12 months, post-treatment	SF-36	Questionnaire score	The QOL did not change significantly between the intervention group and the control group (*p* = 0.59).
(38), Spain		Within 10 years, post-treatment	FACT-B	0–148 points; the higher the score, the higher the quality	PWB (*p* = 0.21), SWB (*p* = 0.40), EWB (*p* = 0.23) FWB (*p* = 0.11), BCS (*p* = 0.39). FACT-B total score (*p* = 0.30) did not improve the QOL.
(72), Denmark		Undergoing treatment	EORTC QLQ-C30	Questionnaire score	Eight of the 15 items were significantly improved: physical functioning (*p* < 0.001), role functioning (*p* < 0.001), emotional functioning (*p* = 0.022), global health status (*p* = 0.017), fatigue (*p* = 0.006), pain (*p* = 0.006), insomnia (*p* = 0.002), and diarrhea (*p* = 0.013).
(45), Canada		Undergoing treatment	SF-36	The higher the score, the better the effect	Significant improvements (*p* < 0.001) for all eight subscales of the survey physical functioning, role physical, bodily pain, general health perception, energy/vitality, social functioning, role emotional and mental health.
(73), Denmark		At least one cycle, post-treatment	SF-36	The higher the score on the questionnaire, the higher the happiness	The intervention had significant effects on 7 of the 10 sub-scales of general wellbeing (*p* < 0.05): physical function (ES = 0.37), role body (ES = 0.37), vitality (ES = 0.55), role emotion (ES = 0.32), mental health (ES = 0.28), body composition scale (ES = 0.35), and mental composition scale (ES = 0.41).
(68), Greece		~3 years, post-treatment	EORTC QLQ-C30	A high score of symptoms indicates a high level of cancer-related symptoms, and a high score of function indicates a good QOL	QOL scores were significantly improved (*p* < 0.05), as well as physical functioning (*p* < 0.05), cognitive functioning (*p* < 0.01), and emotional functioning (*p* < 0.05).
(74), Denmark		At least one cycle, undergoing	EORTC QLQ-C30	Questionnaire score	Significant improvement in QOL (*p* < 0.05).
(59), USA		Post-treatment, time not reported	EORTC QLQ-C30	The higher the score on the function scale, the better the state. The higher the score on the symptom scale, the worse the state	The overall QOL score increased by 8.2 points (*p* = 0.059). There were also significant improvements in physical function (0.08), role function (0.015), and social function (0.031).

FACIT-F, Functional Assessment of Chronic Illness Therapy-Fatigue; FACT-F, Functional Assessment of Cancer Therapy-Fatigue; BFI, Brief Fatigue Inventory; PFS, Piper Fatigue Scale; MFI, Multidimensional Fatigue Inventory; FAQ, Fatigue assessment questionnaire; MFSI-SF, Multidimensional Fatigue Symptom Inventory; FACT-An, Functional Assessment of Cancer Therapy-Anemia Subscale; EORTC QLQ-C30, European Organization for Research and Treatment of Cancer Quality of Life Questionnaire-C30.

A total of 21 studies reported the effect of resistance exercise interventions on the QOL of patients with cancer. The results of seven studies (30, 31, 37, 43, 46, 55, 64) indicated that there was no significant change in the QOL of patients with cancer after exercise interventions compared to the control group. In their study, Simonavice et al. (31) assessed the QOL and psychological wellbeing of patients with breast cancer using the SF-36. The results demonstrated no significant change at baseline, in March, or in June. The best study conducted by Steindorf et al. (37) revealed that following the intervention, the overall QOL in the exercise group exhibited an increase solely in terms of role functioning and pain, with no discernible between the control and excise intervention groups. Ten studies have reported the effect of resistance exercise interventions on participants’ QOL (26–28, 39, 40, 47, 50, 54, 62, 65). Dawson et al. (62) explored the effects of 12 weeks of resistance training and protein supplementation on the body composition in patients with prostate cancer treated with ADT. The results showed improvements in FACT-G and FACT-P scores as well as significant improvements in outcome measures related to QOL. A study by Benton et al. (65) showed that resistance exercise had an effect on improving QOL in both young and old patients with breast cancer. However, the improvement in QOL was relatively more pronounced in young patients with breast cancer, reflecting the differential impact of age on QOL improvement. The other four studies (35, 44, 63, 70) did not directly conclude that resistance exercise improves QOL, but they all showed a marginal improvement in QOL with resistance exercise. Peddle-McIntyre et al. (44) evaluated the effect of progressive resistance exercise on physical function and QOL in 17 patients with lung cancer. The results of SF-36 showed that multiple QOL outcomes had marginal improvement, but the specific cancer QOL (FACT-L) had no change after intervention. Chen et al. (63), in a 4.5-month quasi-experimental study using a single-group longitudinal design, when exploring the effect of elastic band resistance exercise intervention on patients with rectal cancer, found no significant changes in the general QOL assessment scale after the intervention, but a significant decrease in the cancer-specific symptom scale scores. Ammitzboll et al. (70) also demonstrated that the intervention only had favorable effects on emotion and social function. Although other indicators did not reach statistical significance, the results favored the intervention group. 21 studies examined the effects of resistance exercise on QOL in patients with cancer. Of these, 11 studies included patients who had completed cancer treatment at baseline, and 8 studies included patients who were undergoing cancer treatment. Those that completed treatment included three studies on breast cancer, two studies on colorectal cancer, two studies on prostate cancer, one study on head and neck cancer, and three studies on early-stage cancer. Those in treatment included three studies on breast cancer, two studies on prostate cancer, and one study each on head and neck, nasopharyngeal, and colorectal cancer. The remaining two studies included one on pre-treatment intervention for lung cancer and one on breast cancer without any treatment. In the four studies that had completed cancer treatment at the time of the intervention, the majority of findings demonstrated relatively favorable improvements in QOL for patients with cancer who engaged in resistance exercise following the intervention. However, two studies indicated that there were no significant or statistically significant improvements in QOL after the exercise intervention (31, 43). In studies where patients were undergoing cancer treatment at the time of the exercise intervention, the results demonstrated that the majority of studies did not find significant improvements in QOL for patients with cancer following resistance exercise interventions (30, 37, 46, 55, 64). The remaining two studies, Peddle-McIntyre et al. (44) evaluated the feasibility and preliminary efficacy of a PRET intervention for survivors of lung cancer treatment. No significant changes in QOL metrics were observed, with the exception of role fitness, physical pain, and physical health components, which demonstrated significant improvements. Benton et al. (65) sought to assess the impact of age on the QOL of patients with breast cancer following resistance training. Their findings indicated a notable enhancement in the QOL of patients with breast cancer following an exercise intervention, with the younger group exhibiting superior outcomes compared to the older group.

Nine studies have reported the effect of resistance exercise combined with aerobic exercise intervention on the QOL of patients with cancer. The results of two studies indicated that there was no significant difference in overall QOL after the exercise intervention (29, 69). Seven studies reported a significant effect of resistance exercise combined with aerobic exercise on QOL of various types of patients with cancer (25, 48, 52, 56, 60, 67, 71). Dieli-Conwright et al. (60) found that resistance exercise had a significant improvement for both Hispanic and non-Hispanic individuals, especially in social wellbeing, emotional wellbeing, and functional wellbeing. Lei et al. (48) explored the effects of traditional Chinese mind–body medicine exercise on QOL, depression, and anxiety in patients with cancer. On the functional scale, the intervention group showed significant improvement in physical functioning, role functioning, emotional functioning, and social functioning. On the symptom scale, the intervention group showed significant improvement in fatigue, pain, dyspnea, and sleep anxiety. Galvao et al. (56) found significant improvements in the assessment of the general QOL, while there were marginal improvements in the assessment of the cancer-specific QOL, such as physical, emotional, sleep, and pain. Adeline et al. (71), while exploring the effects of a 12-week exercise program on QOL in early-stage elderly patients with cancer, found that the improvement in QOL came primarily from an improvement in the overall state of health, improved physical functioning, and reduced fatigue, whereas the FACT-G assessment showed no significant change. Of these nine studies, five studies involved patients who had completed cancer-related treatment at baseline, while the remaining four studies focused on patients who were currently undergoing cancer treatment. The studies that had completed treatment included four on breast cancer and one on lung cancer. The studies that were in treatment included one on colorectal cancer, one on prostate cancer, and two studies that were not cancer-specific. Nevertheless, it was revealed that only two studies of patients with colorectal and early-stage cancers who were undergoing treatment did not find significant improvements in QOL following resistance exercise combined with aerobic exercise interventions (29, 69).

Ten studies have reported the effect of resistance exercise combined with other exercise interventions on the QOL of patients with various types of cancer. Of these studies, three studies showed little improvement in QOL after exercise intervention (36, 38, 61). Soriano−Maldonado et al. (38) conducted an intervention combining resistance and home exercise in patients with breast cancer. Guided resistance exercise was added twice a week to a home exercise prescription. The results showed that the improvement in patients’ QOL was not ideal. The other seven studies reported significant effects of resistance exercise combined with other exercise interventions on improving QOL (45, 57, 59, 68, 72–74). In these studies, at the start of the exercise intervention, patients with cancer in six studies had completed treatment, patients in three studies were in treatment, and patients in one study contained both. Of the six trials in which cancer-related treatment was completed, three trials in patients with breast cancer and unspecified cancers did not show an improvement in QOL after resistance training combined with other exercise interventions (36, 38, 61). One trial involving patients undergoing treatment, regardless of treatment/recovery stage, had a significant and statistically significant improvement in QOL after the intervention.

#### 3.4.3 Fatigue

**Table 5** shows the changes in the fatigue status of patients with cancer following an exercise intervention in each study experiment. A total of 20 studies examined the effects of resistance exercise intervention on fatigue in patients with cancer.

**Table 5:** Effect of resistance training on fatigue of patients with cancer.

Study	Status	Measurement methods	Descriptive	Main reported outcomes
Resistance exercise				
(43), Belgium	Post-treatment, time not reported	FACIT-F	The score range is 0–52. The higher the score, the lower the fatigue	No significant between-group difference (*p* > 0.05).
(28), China	Post-treatment, time not reported	BFI	Likert scale was used to quantify the degree of fatigue	The fatigue tolerance of TC and RT in both groups was improved (*p* < 0.05), and the fatigue degree in the control group was increased (*p* = 0.01).
(39), USA	>12 months, post-treatment	PFS	Fatigue was evaluated according to the score of the scale. The higher the score, the greater the degree of fatigue	The degree of fatigue decreased by 58% (*p* < 0.01), and no women reported moderate or severe fatigue after RT.
(55), Germany	Undergoing treatment	MFI	The score of general, physical, mental, and motivational activities	There was no significant difference in fatigue changes between the intervention group and the control group (*p* = 0.730).
(62), USA	>3 months, post-treatment	BFI	The higher the score, the more serious the fatigued state	There was no significant difference in fatigue between the two groups (*p* = 0.36), and the exercise group had no significant improvement in fatigue.
(37), Germany	Undergoing treatment	FAQ	The higher the score, the more serious the fatigue	There was a significant difference between the two groups (*p* = 0.044), and the difference between the fatigue groups was the most significant (*p* = 0.013), while emotional fatigue was not significant (*p* = 0.91).
(40),Germany	Undergoing treatment	FAQ	The higher the score, the more serious it is	There was no difference in fatigue between the two groups (*p* = 0.098). Exercise tended to reduce patients’ fatigue, mainly due to physical fatigue (*p* = 0.052), and had no intervention effect on emotional or cognitive fatigue. The effect was more obvious when only considering the patients without depression at baseline, and the difference between groups was 0.039.
(30), Belgium	Undergoing treatment	FACIT-F	The higher the score, the lower the fatigue state	The RT group had the effect of resisting the increase of fatigue, while the fatigue in the control group increased significantly (*p* = 0.009).
(44), Canada	Pre-treatment	FACT-F	Questionnaire score	No significant change in fatigue (0.715).
(50), China	Undergoing treatment	MFSI-SF	Questionnaire score	The degree of fatigue in the exercise group decreased significantly, and the differences between the groups were general fatigue (*p* = 0.035), physical fatigue (*p* = 0.027), emotional fatigue (*p* = 0.044), and mental fatigue (*p* = 0.013), and the total difference was 0.022.
(54), USA	Post-treatment, time not reported	FACIT-F	0–52 points; the higher the score, the lighter the symptoms	Fatigue difference between groups (*p* = 0.006, ES = 2.0).
(70), Denmark	~3 weeks, post-treatment	FACIT-F	The higher the score, the lower the fatigue	There was no significant difference between fatigue groups (*p* = 0.081).
Resistance combined with aerobic exercise				
(29), Australia	Undergoing treatment	MFSI-SF	Scale score	Fatigue level increased after exercise (*p* = 0.28).
(34), USA	Undergoing treatment	MFI-20	Scale 20–100 points; the higher the score, the greater the fatigue	The fatigue score of the exercise group decreased slightly (*p* = 0.10), and the physical fatigue of the exercise group was significantly lower than that of the control group (*p* = 0.036).
(60), USA	Within 6 months, post-treatment	BFI	Scale score	Fatigue was significantly improved (*p* < 0.01).
(69), Denmark	At least one cycle, undergoing treatment	Fact-An	The score on the fatigue scale is 0–52. The higher the score, the lighter the fatigue symptoms	The total score of fatigue in the intervention group was significantly improved after 6 weeks (*p* = 0.002).
(71), Canada	3–12 weeks, undergoing treatment	FACIT-F	The higher the score, the lower the fatigue	After 12 weeks, there was no significant difference between the exercise group and the control group (*p* = 0.09), but the fatigue of the exercise group tended to decrease, and the score increased by 3.5 points.
Resistance combined with other exercises				
(61),Netherlands	Post-treatment, time not reported	MFI	Questionnaire score of 20 statements	General fatigue, reduced activity, mental fatigue, physical fatigue, and reduced motivation (*p* > 0.05).
(38), Spain	Within 10 years, post-treatment	FACT-F	0–52 points; the higher the score, the lower the fatigue	There was no significant difference between the two groups (*p* = 0.07), and there was no improvement in fatigue status.
(73), Denmark	At least one cycle, post-treatment	EORTC-QLQ-C30	Scale score	The fatigue score was reduced in the intervention group by an estimated mean difference of −6.6 points (95% CI −12.3 to −0.9) compared with the control group (*p* = 0.02, effect size = 0.33, 95% CI 0.04 to 0.61).

FACIT-F, Functional Assessment of Chronic Illness Therapy-Fatigue; FACT-F, Functional Assessment of Cancer Therapy-Fatigue; BFI, Brief Fatigue Inventory; PFS, Piper Fatigue Scale; MFI, Multidimensional Fatigue Inventory; FAQ, Fatigue Assessment Questionnaire; MFSI-SF, Multidimensional Fatigue Symptom Inventory; FACT-An, Functional Assessment of Cancer Therapy-Anemia Subscale; EORTC QLQ-C30, European Organization for Research and Treatment of Cancer Quality of Life Questionnaire-C30.

Twelve studies were conducted to examine the effects of resistance exercise interventions on cancer fatigue. Six studies’ results indicated that there was no change or a decrease in fatigue status after the exercise intervention (40, 43, 44, 55, 62, 70), Schmidt et al. (40) conducted a resistance exercise intervention twice a week for 12 weeks in 101 patients with breast cancer. The results showed an increase in total fatigue and physical fatigue during chemotherapy in the control group. Although resistance exercise was found to reduce physical fatigue and improve QOL in patients with cancer to some extent, the observed effects were not statistically significant. The primary factor contributing to the decline in patients’ physical fatigue was a reduction in exercise intensity and frequency, whereas physical fatigue was not associated with emotional and cognitive fatigue and was influenced by the baseline condition of depressed patients. Six studies have reported the positive effects of resistance exercise on reducing fatigue in patients with cancer (28, 30, 37, 39, 50, 54), Cheng et al. (28) explored the effects of TC and resistance training of varying intensities on fatigue and QOL associated with elderly patients with cancer. Following the intervention, fatigue increased in the control group, while fatigue tolerance improved in the TC and resistance training groups, reaching statistically significant levels. Serra et al. (39), while exploring whether resistance training reduces fatigue and decreases systemic and tissue-specific inflammation in patients with breast cancer, found that resistance training reduced cancer-related fatigue by 58%. Steindorf et al. (37) found significant reductions in cancer-related fatigue, but not emotional fatigue, in the exercise group. Hu et al. (50) investigated the benefits of resistance exercise during chemotherapy in patients with nasopharyngeal carcinoma and found that patients in the exercise group had significantly less fatigue, with significant differences in general fatigue (*p* = 0.035), somatic fatigue (*p* = 0.027), emotional fatigue (*p* = 0.044), and mental fatigue (*p* = 0.013). Patients with cancer in six studies had completed cancer treatment, including two studies in breast cancer, one in prostate cancer, one in rectal cancer, and two studies in patients with early-stage cancer that did not differentiate between specific cancers. Patients in five trials are undergoing cancer treatment, including two breast cancer trials, one prostate cancer trial, one head and neck cancer trial, and one gastrointestinal cancer trial. Patients in one other lung cancer study have not yet been treated. The results showed that three studies of patients with cancer who had completed cancer-related treatments showed improvement in fatigue, while three other studies of rectal, breast, and prostate cancer showed no significant improvement in fatigue in patients with cancer with resistance exercise (43, 62, 70). For trials in which subjects were undergoing treatment, the results of two trials showed that resistance exercise did not improve fatigue in patients with cancer (40, 55). Schmidt et al. (40) conducted a resistance exercise intervention in patients with breast cancer; study results showed an increase in total fatigue and physical fatigue during chemotherapy. Exercise tended to reduce patients’ fatigue, mainly due to physical fatigue, and had no intervention effect on emotional or cognitive fatigue. The effect was more obvious when only considering the patients without depression at baseline. In another study of patients with lung cancer who were untreated before the test, the results demonstrated a similar, non-statistically significant improvement in fatigue in patients with cancer following the exercise intervention. Peddle-McIntyre et al. (44) reported the feasibility and preliminary efficacy of a PRET intervention in pre-treatment lung cancer survivors. There were borderline significant improvements in role-physical, bodily pain, and physical health component score. No other fatigue outcomes approached statistical significance, but most changed in a favorable direction.

Five studies reported the effects of resistance exercise combined with aerobic exercise on cancer-related fatigue. Of these five studies, the results of two studies showed little change or even a reduction in cancer-related fatigue after resistance exercise combined with aerobic exercise interventions (29, 71). Singh et al. (29) reported an increase in fatigue associated with the intervention. They explored the feasibility and efficacy of aerobic and resistance training twice a week for 10 weeks during neoadjuvant chemoradiotherapy (CRT) for rectal cancer. Adeline et al. (71) found a trend towards a significant reduction in fatigue and a 3.5-point increase in total fatigue score, although there was no significant change from the control group. Three studies reported significant reductions in fatigue following the intervention (34, 60, 69). The subjects of one study regarding breast cancer had completed cancer treatment. In four other studies, including one with rectal cancer, one with head and neck cancer, and two with unspecified cancers, the subjects were in the cancer treatment phase. The study demonstrated that patients who completed breast cancer-related treatment exhibited statistically significant improvements in fatigue following resistance training combined with aerobic exercise interventions. In studies of undergoing cancer treatment, two studies of patients with head and neck cancer and early-stage cancers did not achieve statistically significant results for improvements in fatigue levels (34, 71). However, three studies did demonstrate some effect on improvements or reductions in cancer-related fatigue. Singh et al. (29) examined the feasibility and preliminary efficacy of a 10-week exercise program in patients with rectal cancer; the results showed an increase in fatigue after intervention.

Three studies have reported the effects of resistance exercise combined with other exercises on fatigue in patients with cancer. Two studies have demonstrated that resistance exercise does not affect fatigue (38, 61). Adamsen et al. (73) evaluated the effects of a multimodal exercise intervention as an adjunct to usual care on fatigue, physical fitness, overall health, physical activity, and QOL in patients with cancer receiving adjuvant chemotherapy or treatment for advanced disease. After 6 weeks of intervention, patients exhibited a significant reduction in fatigue, with a 6.6-point decrease in fatigue scores. The studies included one on breast cancer and two on patients with non-specific cancers. All patients enrolled in the study had completed their cancer treatment prior to the commencement of the exercise intervention. The results of two studies indicated that resistance exercise combined with other exercise interventions did not result in improved fatigue status in patients with cancer.

## 4 Discussion

It is well documented that incorporating exercise into one’s daily routine can promote numerous health benefits, and this also applies to patients with cancer. In addition to enhancing physical health, exercise can assist in the treatment of the side effects of cancer, including physical and psychological changes that can significantly impact muscle strength, QOL, and feelings of fatigue in patients with cancer. This review systematically assessed the effects of resistance exercise, resistance exercise combined with aerobic exercise, and resistance exercise combined with other exercises on physical fitness, QOL, and fatigue in all patients with cancer. Meanwhile, the experimental protocols, types of interventions, frequency of exercise, intensity, duration of exercise, duration of the program, and measures of each outcome indicator varied considerably and were highly heterogeneous across all 48 included studies. Consequently, it is very important to analyze the optimal prescription of resistance exercise interventions that can have a greater positive beneficial effect on the majority of patients with cancer.

We discuss the efficacy of resistance exercise, resistance exercise combined with aerobic exercise, and resistance exercise combined with other exercise interventions for patients with various types of cancer. The primary outcomes of interest are physical fitness, QOL, and fatigue.

In terms of physical fitness, 34 out of 36 studies reported significant effects of resistance exercise on the physical fitness of patients with cancer compared with the control group. Whether the exercise involved resistance exercise training alone, resistance exercise combined with aerobic exercise, or some other form of exercise, all of them significantly improved physical fitness, especially muscle strength, in patients with cancer. Therefore, the adverse effect of reduced muscle mass in patients with cancer undergoing treatment can be effectively addressed through the implementation of these forms of exercise intervention. The impact of resistance exercise on QOL was not particularly prominent; rather, it was found to be most effective when combined with aerobic and other exercises. In the context of clinical cancer treatment, for patients with severely reduced QOL or mental health problems, the development of relevant non-drug intervention program may include a form of intervention that combines resistance exercise with other exercises, with the potential for improved outcomes. In studies of cancer-related fatigue, no significant effect of exercise on fatigue levels has been found. However, the majority of the studies employed a more rigorous intervention protocol, which may have exacerbated the patients’ perception of physical fatigue. This could be a contributing factor to the lack of significant improvement in fatigue levels. Therefore, in the actual treatment process, it is crucial to consider not only the impact on the physical fitness of patients with cancer, but also the potential for other adverse effects of exercise, such as the possibility that higher-intensity exercise may exacerbate the fatigue experienced by patients with cancer.

Owing to symptomatic limitations, the lack of adequate physical activity, and the associated vicious cycle, patients are adversely affected in terms of muscle mass during anticancer treatment. Unless the cancer is eradicated, this can result in a persistent deterioration in physical fitness (75, 76). A series of previous studies have demonstrated that exercise improves physical fitness and QOL in patients with cancer (77, 78), but these studies were small, family-based, and had little control over the intensity and amount of exercise, making it difficult to compare findings. In most sports studies, central location is a significant predictor of increased subject adherence (79). Home-based intervention programs have the potential to enhance patient acceptability while simultaneously reducing the overall cost of program supervision (80). Lam et al. (27) examined whether a 12-month exercise program developed at the start of ADT, which was based on home-based progressive resistance training, reduced adverse effects on body composition, metabolic health, physical functioning, and health-related QOL in patients with prostate cancer. A systematic review concludes that aerobic and resistance exercise improve upper and lower body muscle strength better than traditional care. The study also demonstrated that resistance exercise improved patients’ muscle strength more effectively than aerobic exercise (81). Thus, the impact of enhanced muscular strength may be discernible in interventions for patients with cancer, whether through resistance training alone or in combination with aerobic exercise. A recent meta-analysis also indicates the efficacy of resistance training in alleviating muscle dysfunction in patients with cancer. Based on patient data from 28 trials, it was concluded that exercise significantly improved muscle strength and function (82). Meanwhile, we also found that two of all included studies utilized an intervention model combining anti-resistance with nutritional supplements (51, 62). The result was a significant improvement in physical functioning as well. The principle of optimizing physical function and nutritional supplements in patients with cancer represents a broader concept of rehabilitation that is applicable to all patients with cancer. In patients with incurable cancer, the high prevalence of cachexia means that any intervention measures for this group should take nutritional support and supplementation as an important part of the means (83). Better nutrition is associated with higher baseline nutritional levels and a lower level of systemic inflammation. The effects of interventions to build muscle strength were more pronounced, particularly in terms of muscular endurance and reduced depression. Exercise interventions for patients with cancer during cancer treatment may better maintain or improve physical fitness and mental health outcomes (84), improve cardiorespiratory fitness (85), reduce anxiety and depression (86), improve health-related QOL (16), and reduce cancer-related fatigue (87). In the analysis of the QOL, resistance exercise interventions appear to have a weaker impact on patients with cancer than resistance exercise combined with aerobic and other exercise interventions. Nearly half of the 20 studies on resistance exercise intervention reported no significant effect or even a decrease in QOL, and 4 studies reported only marginal improvements in QOL, with no significant improvement in patients’ QOL (36, 44, 63, 70). However, the effect of resistance exercise intervention alone was not significant compared to resistance exercise combined with aerobic and other exercises. For example, studies by Bloomquist et al. and Nilsen et al. conducted high-intensity heavy resistance exercise interventions in patients with breast and prostate cancer, respectively, but did not elicit positive changes in patients’ QOL (46, 64). Most studies of resistance exercise combined with aerobic and other forms of exercise have demonstrated an improvement in QOL. It is evident that aerobic and other exercises, such as flexibility, stretching, and massage, play a significant role in enhancing QOL. Evidence suggests that patients with cancer suffer from severe psychological burdens, such as anxiety and depression, in addition to degradation of physical functioning from diagnosis to treatment (88), and the relationship between anxiety and depression and cancer prognosis has been well documented (89). The exercise intensity of the aerobic exercise intervention study in this review was approximately 75% HRmax. Resistance combined with stretching, flexibility, balance, and other sports can improve physical fitness. These elements also help to improve body composition and reduce excess adipose tissue and risk factors associated with lack of muscle strength. Additionally, they can reduce chemotherapy-induced neuropathic pain and improve physical and mental QOL (90–92). Resistance exercise, in combination with aerobic and other exercise interventions, represents an effective means of improving QOL and facilitating positive lifestyle changes in patients with cancer.

Fatigue is one of the most common adverse effects of cancer, can be present for years after the end of cancer treatment, and can undermine all aspects of QOL, which is an important factor in reducing survival.

Cancer-related fatigue represents one of the most significant symptoms associated with cancer and its treatment and can have a profound impact on a patient’s QOL. This effect is particularly pronounced during cancer radiotherapy and chemotherapy. From the studies of the effects of resistance exercise on cancer fatigue included in this systematic evaluation, it can be seen that resistance, resistance exercise combined with aerobic, and resistance exercise combined with other exercises did not improve fatigue as much, and that most of the positive effects were concentrated in studies of combined exercise interventions. Furthermore, it is pertinent to highlight that the study by Adamsen et al. employed exercise interventions, including resistance exercise combined with aerobic exercise and relaxation massage. This approach yielded notable outcomes in alleviating fatigue in patients with cancer, in which the role of relaxation massage should not be ignored (73). Reviewing all the included studies, we can see that most of the resistance exercise intensities used were above 75% 1RM, which might be a greater stress load for the participants and dilution of the fatigue-relieving effects. A recent indirect comparative meta-analysis evaluated the effects of diverse types of exercise and other non-pharmacological interventions on cancer-related fatigue during and after cancer treatment. The results showed that relaxation exercises during cancer treatment were the best measure to relieve cancer fatigue, followed by massage, cognitive behavioral therapy combined with physical activity, aerobic and resistance training, resistance training, aerobics, and yoga. At the end of treatment, yoga is the most effective in relieving fatigue, followed by aerobics combined with resistance training, aerobics, and resistance training (93). While most patients prefer low- to moderate-intensity exercise interventions, one study found that moderate- to vigorous-intensity exercise was beneficial for patients with cancer with fatigue compared with low-intensity exercise (94). This is inconsistent with the results of some of the studies included in the review, but there was no control for the amount of exercise, the treatment of patients with cancer involves different methods and phases, and the importance of exercise intensity itself cannot be determined. Therefore, more research comparing the relationship between different exercise intensities and cancer fatigue is needed. Combining the results of the systematic review, it is easy to see that combined exercise interventions for patients with cancer do not result in significant fatigue relief. It is crucial to consider the specific stage and the most appropriate interventions in light of this evidence.

Exercise can enhance motivation to change lifestyle behaviors, improve aerobic fitness and physical function, control fatigue, and enhance QOL. As a non-pharmacological and interventional measure, exercise has been demonstrated to reduce the risk of cancer. Moreover, physical activity plays a beneficial role in numerous cancers during cancer treatment. For instance, the adverse effects associated with cancer treatment can be mitigated, and the efficacy of other treatments can be enhanced, through physical activity. Consequently, in addition to an understanding of the impact of physical activity on various aspects of cancer, it is also necessary to identify the type, amount, and intensity of exercise that has an impact. Currently, cancer treatment employs a combination of therapeutic approaches, tailored to the specific characteristics of the disease, including the type, stage, and progression. These approaches may include targeted therapy, hormonal therapy, radiotherapy, and surgery. Radiotherapy and chemotherapy may induce physiological alterations and adverse effects (95, 96). Common side effects include fatigue, insomnia, nausea, and vomiting. Fatigue is one of the most common side effects of cancer. The utilization of different types and combinations of exercise can be employed for patients with cancer at various stages of treatment. For patients undergoing treatment, resistance training can effectively reduce the side effects of radiotherapy and chemotherapy. For cancer survivors who have completed their treatment, it can strengthen their physical health, enhance their immunity, and reduce the chances of cancer recurrence. The analysis of QOL revealed that resistance training, resistance training combined with aerobic exercise, or other exercises had a significant effect on improving the QOL of patients with cancer, regardless of whether the patients were in the treatment or recovery stage. This indicates that resistance training has a beneficial effect on patients in all stages of cancer treatment. These findings align with previous research indicating that resistance exercise during cancer chemotherapy is safe and may mitigate some of the treatment’s side effects (97). As can be seen from the inclusion in this study of results regarding the QOL of exercise interventions for cancer, the improvement in QOL was mainly due to resistance exercise combined with aerobic exercise as well as other exercises such as home-based exercise intervention models mainly for the treatment or recovery phase of cancer, and the improvement in QOL is more in favor of the population during treatment. Concurrently, the combination of resistance exercise with aerobic or other exercise was found to enhance the quality of life for the majority of cancer patients. There were no comparisons between resistance training and aerobic exercise in the included trials, but a previous randomized trial found no difference in QOL between the two groups by comparing the difference in QOL improvement between resistance and aerobic exercise in patients with cancer, including moderate to high intensity and home training (98). When resistance training is combined with aerobic exercise in interventions for patients with cancer undergoing treatment, the improvements in QOL may be greater than if the interventions were performed alone. As mentioned earlier, there was no consistent improvement in fatigue in patients with cancer regardless of the type of exercise intervention or stage of cancer treatment. Singh et al. (29), while exploring the effect of resistance exercise combined with aerobic exercise on the QOL of patients with breast cancer undergoing treatment, found that the patients showed increased fatigue. This may be related to the prevalence of fatigue in patients with most types of cancer. Patients often experience both psychological and physical stress during cancer treatment, and the increase in cancer-related fatigue is more severe, more painful, and less likely to be relieved by rest than fatigue in the healthy population. Although the exact etiology of cancer-related fatigue is not fully understood, it is thought to be based on the physical and psychological effects of cancer treatment. There are many theories about the factors that contribute to cancer-related fatigue. For example, one study suggests that the reduction in blood cells caused by chemotherapy may lead to anemia, which may lead to fatigue (99, 100). Meanwhile, treatment-induced activation of pro-inflammatory cytokines may be one trigger (101). Other factors include medications, psychological distress, altered immune function, excessive inactivity, neuromuscular dysfunction, and cognitive factors. It has already been mentioned that relaxation and massage are effective interventions during cancer treatment (73), but the role of relaxation after cancer treatment is less significant, suggesting that strategies to effectively manage fatigue during cancer treatment should include relaxation sessions in addition to individualized exercise and other non-pharmacological interventions. However, the effectiveness of relaxation massage declines after cancer treatment. More time should be spent on interventions to increase physical activity. Yoga is beneficial during and after cancer treatment, as are aerobic exercise, resistance training, and combined aerobic and resistance training. Only the magnitude of the effect is slightly lower.

Although there is sufficient evidence to support physical activity and exercise in adult patients with cancer, it is safe and acceptable for patients with all cancer types to engage in physical activity and exercise before, during, and after cancer treatment (102, 103). Designing a rational and scientific exercise intervention program for patients with cancer can better counteract the many side effects of the treatment process, better serve the rehabilitation program, improve the physical function of patients with cancer, reduce the feeling of fatigue, and, at the same time, improve the overall QOL. The majority of current studies have been conducted during and immediately after the treatment of patients with breast cancer. In contrast, relatively few studies on lung, digestive, and prostate cancers have been conducted. However, the evidence-based research literature in this area is rapidly accumulating. This study has several notable strengths. Primarily, it does not focus on a particular type of cancer, but rather examines all eligible cancer types, which makes it more broadly applicable. Second, a multilevel distinction was made in the form of exercise to facilitate the observation of the specific interventions in the future. Finally, the latest research evidence was included in the collection of research data for qualitative analysis. It should be noted that the study does have some limitations. Firstly, the literature collection is somewhat limited. A smaller number of studies have been conducted for specific cancers, and the findings may be biased. Second, owing to the heterogeneity of the measurement of the study results, a data-supported meta-analysis could not be performed, which could only provide a relatively limited reference value for the prognosis of clinical cancer treatment.

## 5 Conclusions

This systematic review shows that resistance exercise, resistance exercise combined with aerobic exercise, and resistance exercise combined with other exercises have a favorable intervention effect on the physical fitness (muscle strength, cardiorespiratory fitness, etc.) and QOL in patients with cancer. As far as physical fitness is concerned, all kinds of exercise interventions are effective in improving it, regardless of the patient’s stage of cancer treatment, but resistance is the best. QOL improvements, on the other hand, tend to favor a relative combination of resistance and aerobic and other exercises. The effects of the three resistance methods on alleviating cancer fatigue are inconsistent and controversial. Our conclusions may provide some valuable references for clinical cancer non-pharmacological intervention treatment and rehabilitation.

## Data availability statement

The raw data supporting the conclusions of this article will be made available by the authors, without undue reservation.

## Author contributions

QZ: Methodology, Software, Writing – original draft. YG: Methodology, Software, Writing – original draft. WW: Project administration, Software, Writing – review & editing. XZ: Writing – review & editing. JY: Writing – review & editing. HH: Formal analysis, Methodology, Software, Writing – review & editing.
