# Supplementary material for: Effect of resistance exercise on physical fitness, quality of life, and fatigue in patients with cancer: a systematic review
Source: Front Oncol. 2024 Jul 19;14:1393902. doi: 10.3389/fonc.2024.1393902 (PMC11294253; doi:10.3389/fonc.2024.1393902)
Supplement: Supplementary file 1 [file Table_1.docx]

**Appendix Table 1** Systematic literature review search terms and strategy

| **Search terms for PubMed** |
| --- |
| **#1**("Neoplasms"[MeSH Terms] OR "Tumor"[Title/Abstract] OR "Neoplasm"[Title/Abstract] OR "Tumors"[Title/Abstract] OR "Neoplasia"[Title/Abstract] OR "Neoplasias"[Title/Abstract] OR "Cancer"[Title/Abstract] OR "Cancers"[Title/Abstract] OR "malignant neoplasm"[Title/Abstract] OR "Malignancy"[Title/Abstract] OR "Malignancies"[Title/Abstract] OR "malignant neoplasms"[Title/Abstract] OR "neoplasm malignant"[Title/Abstract] OR "neoplasms malignant"[Title/Abstract] OR "benign neoplasms"[Title/Abstract] OR "benign neoplasm"[Title/Abstract] OR "neoplasms benign"[Title/Abstract] OR "neoplasm benign"[Title/Abstract]) |
| #2 ("Resistance Training "[MeSH Terms] OR " Training, Resistance "[Title/Abstract] OR " Strength Training "[Title/Abstract] OR " Weight-Lifting Strengthening Program "[Title/Abstract] OR " Strengthening Programs "[Title/Abstract] OR " Exercise Program "[Title/Abstract]) |
| **#1AND#2** |
| **Search terms for Web of science** |
| TS= ((neoplasms OR tumor OR neoplasia OR cancer OR malignant neoplasm OR malignancy OR malignant OR neoplasm malignant OR benign neoplasms OR neoplasm benign) AND (Resistance Training OR Training, Resistance OR Strength Training OR Weight-Lifting Strengthening Program OR Strengthening Programs OR Exercise Program) |
| **Search terms for Scopus** |
| **#1** TITLE-ABS-KEY ("neoplasms" OR " tumor" OR " neoplasia" OR " cancer" OR " malignant neoplasm" OR " malignancy" OR " malignant" OR " neoplasm malignant" OR " benign neoplasms" OR " neoplasm benign") |
| **#2** TITLE-ABS-KEY (Resistance Training OR Training, Resistance OR Strength Training) |
| **#1 AND #2** |
