# Supplementary material for: Effect of resistance exercise on physical fitness, quality of life, and fatigue in patients with cancer: a systematic review
Source: Front Oncol. 2024 Jul 19;14:1393902. doi: 10.3389/fonc.2024.1393902 (PMC11294253; doi:10.3389/fonc.2024.1393902)
Supplement: Supplementary file 2 [file Table_2.docx]

**Appendix Table2** The reference list of the 48 included studies

| **First author, publication year** | I**nformation of included studies** |
| --- | --- |
| Adamsen et al, 2003 | Adamsen L, Midtgaard J, Rorth M, et al. Feasibility, physical capacity, and health benefits of a multidimensional exercise program for cancer patients undergoing chemotherapy. *Support Care Cancer*. 2003;11(11):707-716. doi:10.1007/s00520-003-0504-2 |
| Adamsen et al 2006 | Adamsen L, Quist M, Midtgaard J, et al. The effect of a multidimensional exercise intervention on physical capacity, well-being and quality of life in cancer patients undergoing chemotherapy. *Support Care Cancer*. 2006;14(2):116-127. doi:10.1007/s00520-005-0864-x |
| Herrero et al 2006 | Herrero F, San Juan AF, Fleck SJ, et al. Combined aerobic and resistance training in breast cancer survivors: A randomized, controlled pilot trial. *Int J Sports Med*. 2006;27(7):573-580. doi:10.1055/s-2005-865848 |
| Quist et al 2006 | Quist M, Rorth M, Zacho M, et al. High-intensity resistance and cardiovascular training improve physical capacity in cancer patients undergoing chemotherapy. *Scand J Med Sci Sports*. 2006;16(5):349-357. doi:10.1111/j.1600-0838.2005.00503.x |
| Battaglini et al 2007 | Battaglini C, Bottaro M, Dennehy C, et al. The effects of an individualized exercise intervention on body composition in breast cancer patients undergoing treatment [published correction appears in Sao Paulo Med J. 2007 Sep 6;125(5):303. Hackney, Anthony [corrected to Hackney, Anthony Carl]]. *Sao Paulo Med J*. 2007;125(1):22-28. doi:10.1590/s1516-31802007000100005 |
| Backer et al 2008 | De Backer IC, Vreugdenhil G, Nijziel MR, Kester AD, van Breda E, Schep G. Long-term follow-up after cancer rehabilitation using high-intensity resistance training: persistent improvement of physical performance and quality of life. *Br J Cancer*. 2008;99(1):30-36. doi:10.1038/sj.bjc.6604433 |
| Adamsen et al 2009 | Adamsen L, Quist M, Andersen C, et al. Effect of a multimodal high intensity exercise intervention in cancer patients undergoing chemotherapy: randomised controlled trial. *BMJ*. 2009;339:b3410. Published 2009 Oct 13. doi:10.1136/bmj.b3410 |
| Galvão et al 2010 | Galvão DA, Taaffe DR, Spry N, Joseph D, Newton RU. Combined resistance and aerobic exercise program reverses muscle loss in men undergoing androgen suppression therapy for prostate cancer without bone metastases: a randomized controlled trial. *J Clin Oncol*. 2010;28(2):340-347. doi:10.1200/JCO.2009.23.2488 |
| Noble et al 2012 | Noble M, Russell C, Kraemer L, Sharratt M. UW WELL-FIT: the impact of supervised exercise programs on physical capacity and quality of life in individuals receiving treatment for cancer. *Support Care Cancer*. 2012;20(4):865-873. doi:10.1007/s00520-011-1175-z |
| Peddle-McIntyre et al 2012 | Peddle-McIntyre CJ, Bell G, Fenton D, McCargar L, Courneya KS. Feasibility and preliminary efficacy of progressive resistance exercise training in lung cancer survivors. *Lung Cancer*. 2012;75(1):126-132. doi:10.1016/j.lungcan.2011.05.026 |
| Winters-Stone et al 2012 | Winters-Stone KM, Dobek J, Bennett JA, Nail LM, Leo MC, Schwartz A. The effect of resistance training on muscle strength and physical function in older, postmenopausal breast cancer survivors: a randomized controlled trial. *J Cancer Surviv*. 2012;6(2):189-199. doi:10.1007/s11764-011-0210-x |
| Andersen et al 2013 | Andersen C, Rørth M, Ejlertsen B, et al. The effects of a six-week supervised multimodal exercise intervention during chemotherapy on cancer-related fatigue. *Eur J Oncol Nurs*. 2013;17(3):331-339. doi:10.1016/j.ejon.2012.09.003 |
| Lønbro et al 2013 | Lønbro S, Dalgas U, Primdahl H, et al. Progressive resistance training rebuilds lean body mass in head and neck cancer patients after radiotherapy--results from the randomized DAHANCA 25B trial. *Radiother Oncol*. 2013;108(2):314-319. doi:10.1016/j.radonc.2013.07.002 |
| Benton et al 2014 | Benton MJ, Schlairet MC, Gibson DR. Change in quality of life among breast cancer survivors after resistance training: is there an effect of age?. *J Aging Phys Act*. 2014;22(2):178-185. doi:10.1123/japa.2012-0227 |
| Steindorf et al 2014 | Steindorf K, Schmidt ME, Klassen O, et al. Randomized, controlled trial of resistance training in breast cancer patients receiving adjuvant radiotherapy: results on cancer-related fatigue and quality of life. *Ann Oncol*. 2014;25(11):2237-2243. doi:10.1093/annonc/mdu374 |
| Casla et al 2015 | Casla S, López-Tarruella S, Jerez Y, et al. Supervised physical exercise improves VO2max, quality of life, and health in early-stage breast cancer patients: a randomized controlled trial. *Breast Cancer Res Treat*. 2015;153(2):371-382. doi:10.1007/s10549-015-3541-x |
| Foley et al 2015 | Foley MP, Barnes VA, Hasson SM. Effects of a community-based multimodal exercise program on physical function and quality of life in cancer survivors: a pilot study. *Physiother Theory Pract*. 2015;31(5):303-312. doi:10.3109/09593985.2015.1004390 |
| Nilsen et al 2015 | Nilsen TS, Raastad T, Skovlund E, et al. Effects of strength training on body composition, physical functioning, and quality of life in prostate cancer patients during androgen deprivation therapy. *Acta Oncol*. 2015;54(10):1805-1813. doi:10.3109/0284186X.2015.1037008 |
| Schmidt et al 2015 | Schmidt ME, Wiskemann J, Armbrust P, Schneeweiss A, Ulrich CM, Steindorf K. Effects of resistance exercise on fatigue and quality of life in breast cancer patients undergoing adjuvant chemotherapy: A randomized controlled trial. *Int J Cancer*. 2015;137(2):471-480. doi:10.1002/ijc.29383 |
| Simonavice et al 2015 | Simonavice E, Liu PY, Ilich JZ, Kim JS, Arjmandi BH, Panton LB. The Effects of Resistance Training on Physical Function and Quality of Life in Breast Cancer Survivors. *Healthcare (Basel)*. 2015;3(3):695-709. Published 2015 Aug 11. doi:10.3390/healthcare3030695 |
| Hagstrom et al 2016 | Hagstrom AD, Marshall PW, Lonsdale C, Cheema BS, Fiatarone Singh MA, Green S. Resistance training improves fatigue and quality of life in previously sedentary breast cancer survivors: a randomised controlled trial. *Eur J Cancer Care (Engl)*. 2016;25(5):784-794. doi:10.1111/ecc.12422 |
| Dawson et al 2018 | Dawson JK, Dorff TB, Todd Schroeder E, Lane CJ, Gross ME, Dieli-Conwright CM. Impact of resistance training on body composition and metabolic syndrome variables during androgen deprivation therapy for prostate cancer: a pilot randomized controlled trial. *BMC Cancer*. 2018;18(1):368. Published 2018 Apr 3. doi:10.1186/s12885-018-4306-9 |
| Foley et al 2018 | Foley MP, Hasson SM, Kendall E. Effects of a Translational Community-Based Multimodal Exercise Program on Quality of Life and the Influence of Start Delay on Physical Function and Quality of Life in Breast Cancer Survivors: A Pilot Study. *Integr Cancer Ther*. 2018;17(2):337-349. doi:10.1177/1534735417731514 |
| Grote et al 2018 | Grote M, Maihöfer C, Weigl M, Davies-Knorr P, Belka C. Progressive resistance training in cachectic head and neck cancer patients undergoing radiotherapy: a randomized controlled pilot feasibility trial. *Radiat Oncol*. 2018;13(1):215. Published 2018 Nov 6. doi:10.1186/s13014-018-1157-0 |
| Hausmann et al 2018 | Hausmann F, Iversen VV, Kristoffersen M, Gundersen H, Johannsson E, Vika M. Combined aerobic and resistance training improves physical capacity in women treated for gynecological cancer. *Support Care Cancer*. 2018;26(10):3389-3396. doi:10.1007/s00520-018-4185-2 |
| Serra et al 2018 | Serra MC, Ryan AS, Ortmeyer HK, Addison O, Goldberg AP. Resistance training reduces inflammation and fatigue and improves physical function in older breast cancer survivors. *Menopause*. 2018;25(2):211-216. doi:10.1097/GME.0000000000000969 |
| Singh et al 2018 | Singh F, Galvão DA, Newton RU, Spry NA, Baker MK, Taaffe DR. Feasibility and Preliminary Efficacy of a 10-Week Resistance and Aerobic Exercise Intervention During Neoadjuvant Chemoradiation Treatment in Rectal Cancer Patients. *Integr Cancer Ther*. 2018;17(3):952-959. doi:10.1177/1534735418781736 |
| Ammitzbøll et al 2019 | Ammitzbøll G, Kristina Kjær T, Johansen C, et al. Effect of progressive resistance training on health-related quality of life in the first year after breast cancer surgery - results from a randomized controlled trial. *Acta Oncol*. 2019;58(5):665-672. doi:10.1080/0284186X.2018.1563718 |
| Bloomquist et al 2019 | Bloomquist K, Adamsen L, Hayes SC, et al. Heavy-load resistance exercise during chemotherapy in physically inactive breast cancer survivors at risk for lymphedema: a randomized trial. *Acta Oncol*. 2019;58(12):1667-1675. doi:10.1080/0284186X.2019.1643916 |
| Santos et al 2019 | Santos WDND, Vieira A, de Lira CAB, et al. Once a Week Resistance Training Improves Muscular Strength in Breast Cancer Survivors: A Randomized Controlled Trial. *Integr Cancer Ther*. 2019; 18:1534735419879748. doi:10.1177/1534735419879748 |
| Chen et al 2020 | Chen SC, Huang HP, Huang WS, et al. Non-randomized preliminary study of an education and elastic-band resistance exercise program on severity of neuropathy, physical function, muscle strength and endurance & quality of life in colorectal cancer patients experiencing oxaliplatin-induced peripheral neuropathy. *Eur J Oncol Nurs*. 2020;49:101834. doi:10.1016/j.ejon.2020.101834 |
| Felser et al 2020 | Felser S, Behrens M, Liese J, et al. Feasibility and Effects of a Supervised Exercise Program Suitable for Independent Training at Home on Physical Function and Quality of Life in Head and Neck Cancer Patients: A Pilot Study. *Integr Cancer Ther*. 2020;19:1534735420918935. doi:10.1177/1534735420918935 |
| Hong et al 2020 | Hong Y, Wu C, Wu B. Effects of Resistance Exercise on Symptoms, Physical Function, and Quality of Life in Gastrointestinal Cancer Patients Undergoing Chemotherapy. *Integr Cancer Ther*. 2020;19:1534735420954912. doi:10.1177/1534735420954912 |
| Lam et al 2020 | Lam T, Cheema B, Hayden A, et al. Androgen deprivation in prostate cancer: benefits of home-based resistance training. *Sports Med Open*. 2020;6(1):59. Published 2020 Dec 14. doi:10.1186/s40798-020-00288-1 |
| Xiao et al 2020 | Xiao C, Beitler JJ, Higgins KA, et al. Pilot study of combined aerobic and resistance exercise on fatigue for patients with head and neck cancer: Inflammatory and epigenetic changes. *Brain Behav Immun*. 2020;88:184-192. doi:10.1016/j.bbi.2020.04.044 |
| Adeline et al 2021 | Adeline F, Hugo PR, René M, Tàmàs F, Eléonor R, Michel P. Effects of a mixed exercise program on cancer related-fatigue and health-related quality of life in oncogeriatric patients: A feasibility study [published correction appears in J Geriatr Oncol. 2022 Sep;13(7):1070]. *J Geriatr Oncol*. 2021;12(6):915-921. doi:10.1016/j.jgo.2021.02.025 |
| Aydin et al 2021 | Aydin M, Kose E, Odabas I, Meric Bingul B, Demirci D, Aydin Z. The Effect of Exercise on Life Quality and Depression Levels of Breast Cancer Patients. *Asian Pac J Cancer Prev*. 2021;22(3):725-732. Published 2021 Mar 1. doi:10.31557/APJCP.2021.22.3.725 |
| Cheng et al 2021 | Cheng D, Wang X, Hu J, et al. Effect of Tai Chi and Resistance Training on Cancer-Related Fatigue and Quality of Life in Middle-Aged and Elderly Cancer Patients. *Chin J Integr Med*. 2021;27(4):265-272. doi:10.1007/s11655-021-3278-9 |
| Dieli-Conwright et al 2021 | Dieli-Conwright CM, Fox FS, Tripathy D, et al. Hispanic ethnicity as a moderator of the effects of aerobic and resistance exercise on physical fitness and quality-of-life in breast cancer survivors. *J Cancer Surviv*. 2021;15(1):127-139. doi:10.1007/s11764-020-00918-3 |
| Hu et al 2021 | Hu Q, Zhao D. Effects of resistance exercise on complications, cancer-related fatigue and quality of life in nasopharyngeal carcinoma patients undergoing chemoradiotherapy: A randomised controlled trial. *Eur J Cancer Care (Engl)*. 2021;30(1):e13355. doi:10.1111/ecc.13355 |
| Piraux et al 2021 | Piraux E, Caty G, Renard L, et al. Effects of high-intensity interval training compared with resistance training in prostate cancer patients undergoing radiotherapy: a randomized controlled trial. *Prostate Cancer Prostatic Dis*. 2021;24(1):156-165. doi:10.1038/s41391-020-0259-6 |
| Lee et al 2022 | Lee KJ, An KO. Impact of High-Intensity Circuit Resistance Exercise on Physical Fitness, Inflammation, and Immune Cells in Female Breast Cancer Survivors: A Randomized Control Trial. *Int J Environ Res Public Health*. 2022;19(9):5463. Published 2022 Apr 29. doi:10.3390/ijerph19095463 |
| Lei et al 2022 | Lei J, Yang J, Dong L, et al. An exercise prescription for patients with lung cancer improves the quality of life, depression, and anxiety. *Front Public Health*. 2022;10:1050471. Published 2022 Nov 17. doi:10.3389/fpubh.2022.1050471 |
| Piraux et al 2022 | Piraux E, Reychler G, Vancraeynest D, Geets X, Léonard D, Caty G. High-intensity aerobic interval training and resistance training are feasible in rectal cancer patients undergoing chemoradiotherapy: a feasibility randomized controlled study. *Rep Pract Oncol Radiother*. 2022;27(2):198-208. Published 2022 May 19. doi:10.5603/RPOR.a2022.0036 |
| Soriano-Maldonado et al 2022 | Soriano-Maldonado A, Díez-Fernández DM, Esteban-Simón A, et al. Effects of a 12-week supervised resistance training program, combined with home-based physical activity, on physical fitness and quality of life in female breast cancer survivors: the EFICAN randomized controlled trial. *J Cancer Surviv*. 2023;17(5):1371-1385. doi:10.1007/s11764-022-01192-1 |
| Winters-Stone et al 2022 | Winters-Stone KM, Torgrimson-Ojerio B, Dieckmann NF, Stoyles S, Mitri Z, Luoh SW. A randomized-controlled trial comparing supervised aerobic training to resistance training followed by unsupervised exercise on physical functioning in older breast cancer survivors. *J Geriatr Oncol*. 2022;13(2):152-160. doi:10.1016/j.jgo.2021.08.003 |
| Andrioti et al 2023 | Andrioti A, Papadopetraki A, Maridaki M, Philippou A. The Effect of a Home-Based Tele-Exercise Training Program on the Quality of Life and Physical Performance in Breast Cancer Survivors. *Sports (Basel)*. 2023;11(5):102. Published 2023 May 10. doi:10.3390/sports11050102 |
| Houben et al 2023 | Houben LHP, Overkamp M, VAN Kraaij P, et al. Resistance Exercise Training Increases Muscle Mass and Strength in Prostate Cancer Patients on Androgen Deprivation Therapy. *Med Sci Sports Exerc*. 2023;55(4):614-624. doi:10.1249/MSS.0000000000003095 |
